# Multifunctional SnO_2_ QDs/MXene Heterostructures as Laminar Interlayers for Improved Polysulfide Conversion and Lithium Plating Behavior

**DOI:** 10.1007/s40820-024-01446-w

**Published:** 2024-06-28

**Authors:** Shungui Deng, Weiwei Sun, Jiawei Tang, Mohammad Jafarpour, Frank Nüesch, Jakob Heier, Chuanfang Zhang

**Affiliations:** 1 College of Materials Science and Engineering, Sichuan University, Chengdu, 610065 People’s Republic of China; 2 Laboratory for Functional Polymers, Swiss Federal Laboratories for Materials Science and Technology (EMPA), Überlandstrasse 129, 8600 Dübendorf, Switzerland; 3 Institute of Materials Science and Engineering, Ecole Polytechnique Fédérale de Lausanne (EPFL), Station 12, 1015 Lausanne, Switzerland; 4 Key Laboratory of Quantum Materials and Devices of Ministry of Education, School of Physics, Southeast University, Nanjing, 211189 People’s Republic of China; 5 SEU-FEI Nano-Pico Center, Key Laboratory of MEMS of Ministry of Education, Southeast University, Nanjing, 210096 People’s Republic of China

**Keywords:** Lithium–sulfur battery, Heterogeneous catalysis, Heterostructure, Redox kinetics, Lithium dendrites

## Abstract

Poor cycling stability in lithium–sulfur (Li–S) batteries necessitates advanced electrode/electrolyte design and innovative interlayer architectures. Heterogeneous catalysis has emerged as a promising approach, leveraging the adsorption and catalytic performance on lithium polysulfides (LiPSs) to inhibit LiPSs shuttling and improve redox kinetics. In this study, we report an ultrathin and laminar SnO_2_@MXene heterostructure interlayer (SnO_2_@MX), where SnO_2_ quantum dots (QDs) are uniformly distributed across the MXene layer. The combined structure of SnO_2_ QDs and MXene, along with the creation of numerous active boundary sites with coordination electron environments, plays a critical role in manipulating the catalytic kinetics of sulfur species. The Li–S cell with the SnO_2_@MX-modified separator not only demonstrates superior electrochemical performance compared to cells with a bare separator but also induces homogeneous Li deposition during cycling. As a result, an areal capacity of 7.6 mAh cm^−2^ under a sulfur loading of 7.5 mg cm^−2^ and a high stability over 500 cycles are achieved. Our work demonstrates a feasible strategy of utilizing a laminar separator interlayer for advanced Li–S batteries awaiting commercialization and may shed light on the understanding of heterostructure catalysis with enhanced reaction kinetics.

**Supplementary Information:**

The online version contains supplementary material available at 10.1007/s40820-024-01446-w.

## Highlights

- The interfacing between SnO_2_ and MXene alters electronic structures, shifting the *d*-band center in transition metals, enhancing catalytic efficiency by reducing electron filling in antibonding orbitals.
- A binder-free, ultrathin, laminar heterostructured interlayer on polypropylene separator is demonstrated. The ionic sieving mechanism and efficient adsorption–catalysis process enable deeper charge/discharge cycle and improved stability.
- The improved catalytic conversion and suppressed lithium dendrites formation enable a high loading of 7.5 mg cm^−2^ and an initial area capacity of 7.6 mAh cm^−2^.

**Supplementary Information:**

The online version contains supplementary material available at 10.1007/s40820-024-01446-w.

## Graphical abstract

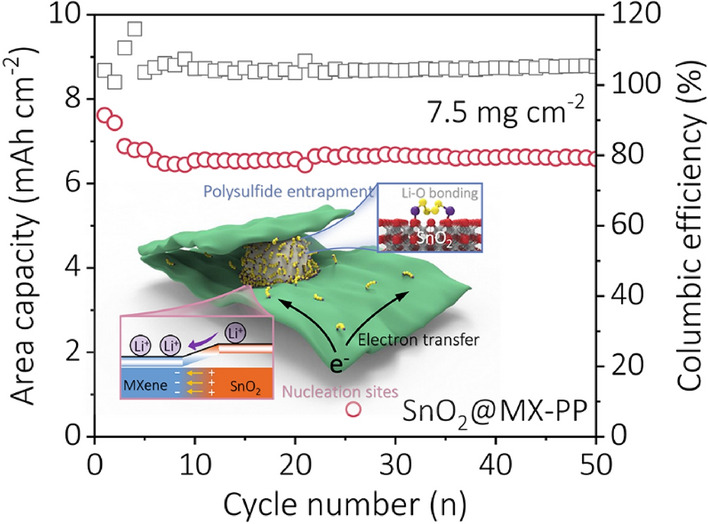

## Introduction

Lithium–sulfur (Li–S) batteries, with their high theoretical specific capacity (1675 mAh g^−1^), eco-friendliness, and abundant availability of S, hold great promise as next-generation energy storage technology [1–3]. However, their commercialization faces challenges pertaining to both cathode and anode. In particular, the S cathode exhibits inherent limitations such as sluggish redox kinetics, dissolution of intermediate lithium polysulfides (LiPSs), and the shuttle effect, which result in limited discharge depth and poor cyclic stability [4, 5]. On the other hand, safety concerns arise from the dendrite growth observed at the Li anode [6, 7] and/or from Li corrosion [8, 9]. Addressing these challenges is crucial for the successful commercialization of Li–S batteries and unlocking their full potential as advanced energy storage systems.

The design of an efficient separator interlayer has been proved effective in mitigating the shuttle effect and suppressing the formation of Li dendrites [10–12]. Compared with sulfur cathode modification, the separator strategy allows limited dissolution of polysulfides but restricts their migration to the cathode side. By tuning the permeability of the separator to selectively transport Li ions while blocking anions (i.e., polysulfides), dissolved LiPSs are retained on the cathode side, thus prevent their shuttling [13, 14]. In particular, by designing an elaborated interlayer with strong adsorption and high catalytic activity, the charge transfer and LiPSs redox kinetics can be significantly improved [15, 16]. In addition, interlayers featuring lithiophilic sites and a high Young’s modulus on the anode side are harnessed to homogenize Li ion flux and restrict the growth of Li dendrites during the charging process [17, 18]. Recent studies have demonstrated the effectiveness of dual-function separator interlayers with both LiPSs redox kinetics enhancement and Li dendrite inhibition [19, 20]. These interlayers are typically composed of carbon or metal compounds with diverse structures, such as porous carbon networks [21], hollow structures [22, 23], and hierarchical nanosheets [24–26]. It is worth noting that, these interlayer frameworks show high porosity and exhibit ~ µm thickness. However, excessive porosity of the interlayer is undesirable as it requires more electrolyte to fill the pores, thereby compromising the overall cell energy density [27]. Besides, the presence of inactive components, i.e., the separator interlayer, often referred to as “dead weight,” is an inevitable consequence that should also be taken into consideration [28]. Alternatively, the laminar structure, formed by the self-stacking of two-dimensional (2D) nanosheets, provides selective channels within the empty voids among neighboring nanosheets in both in-plane and out-of-plane directions, resulting in a dense framework and preventing the formation of large pores [29, 30]. Hence, an ultrathin laminar interlayer, combining permeability and selectivity, while featuring low porosity and negligible weight, is of significant promise.

MXene is an emerging 2D material with high potential in energy conversion and storage applications [31, 32]. The material is renowned for its exceptional conductivity (in Ti_3_C_2_T_*x*_ MXene), atomic-scale thickness, and abundant terminal functional groups such as –OH, –O, and –F [33]. These functional groups play a crucial role in anchoring LiPSs and accelerating their catalytic conversion in Li–S batteries [34]. However, since the functional groups provide the main adsorption sites for LiPSs, the binding strength is relatively weak, resulting in a limited catalytic activity [35]. Tuning the electronic states of MXene can potentially promote the intrinsic catalytic properties from the inert MXene surfaces [36, 37]. Modulating the electronic structure through constructing a Mott–Schottky heterostructure represents an effective method, thereby inducing an oriented and strong internal electric field [38, 39]. The altered electronic structure on both sides of the heterojunction leads to strong chemical adsorption and highly-efficient catalytic effects for LiPSs [40, 41]. For instance, Sun et al. fabricated a Mott–Schottky heterostructure by encapsulating metallic Co nanoparticles in N-doped carbon [42]. They revealed that the redistribution of charge at the heterojunction can propel Li ion mobility, enhance LiPSs immobilization, and reduce the reaction energy barrier. Therefore, engineering heterostructures with a rational structural design holds promise as an effective strategy for achieving high-performance Li–S batteries.

Herein, we present the design of an ultrathin and laminar separator interlayer for Li–S batteries, utilizing a 0D-2D SnO_2_ quantum dots (QDs)/MXene heterostructure (SnO_2_@MX). Such novel heterostructure combines the highly polar SnO_2_, conductive MXene, and active heterojunctions, synergistically enabling strong anchoring of LiPSs, rapid electron/ion transportation, and efficient catalytic conversion. By introducing the optimized SnO_2_@MX separator interlayer, we successfully enhance the conversion kinetics while effectively suppressing the shuttle effect and inhibiting Li dendrite growth. The fabricated Li–S cell with the SnO_2_@MX separator interlayer demonstrates excellent electrochemical performances. It achieves a high initial capacity of > 1400 mAh g^−1^ at 0.05 °C, 845 mAh g^−1^ at 2 °C, and a low-capacity decay of only 0.052% per cycle over 500 cycles. Furthermore, under a high sulfur loading of 7.5 mg cm^−2^, a high initial areal capacity of 7.6 mAh cm^−2^ with a decent stability can be obtained. These findings pave new ways for further advancements in the field of Li–S batteries and highlight the potential of heterostructured catalysts for high-performance energy storage applications.

## Experimental Section

### Synthesis of MXene

The Ti_3_AlC_2_ MAX (hexagonal carbides and nitrides with general formula M_*n*+1_AX_*n*_) phase (Laizhou Kai Kai Ceramic Materials Co., Ltd.) was selectively etched using a minimally intensive layer delamination (MILD) synthesis method to produce Ti_3_C_2_T_*x*_ MXene. Typically, 3.2 g of lithium fluoride (LiF, Sigma-Aldrich) was dissolved in 40 mL, 9 M hydrochloric acid (HCl, 37%, VWR) as the etching solution. Subsequently, 2 g of MAX phase was slowly added to above etching solution under vigorously stirring. The etching process was conducted for 48 h at 50 °C. After etching, the mixture was transferred to centrifuge tubes and centrifuged at 1500 rcf for 5 min. The supernatant was decanted, and the sediment was washed with 40 mL of ultrapure water. The washing step was repeated 5 times, each time centrifuging for 5 min at 1500 rcf, until the pH of the supernatant reached approximately 6. The obtained suspension was vigorously shaken using a vortex machine for 30 min. Then, the mixture was subjected to ultrasound treatment for 1 h under an ice bath with Ar bubbling to further delaminate into few-layered or single-layered MXene. After centrifuging the mixture at 1500 rcf for 30 min, the fully delaminated MXene nanosheets were obtained in the supernatant. The supernatant was then further centrifuged at 15,000 rcf for another 30 min to collect the MXene nanosheets.

### Synthesis of SnO_2_@MX, 0.5-SnO_2_@MX, 2-SnO_2_@MX, and H-MX

For the synthesis of SnO_2_@MX, typically, 180 mg of tin chloride hydrate (SnCl_4_⋅5H_2_O, 98%, Sigma-Aldrich) was dissolved in 20 mL of ultrapure water. The pH of the solution was adjusted to approximately 8 with ammonium hydroxide (NH_3_⋅H_2_O, 25% ~ 28%, Fluka). The above solution was then slowly added into 80 mL Ti_3_C_2_T_*x*_ MXene colloidal solution (1 mg mL^−1^) while vigorously stirred and ultrasonicated under ice bath separately for 60 min. Subsequently, the mixture was transferred to a Teflon-lined stainless-steel autoclave, heated to 120 °C, and maintained at this temperature for 6 h. After cooling down to room temperature, the SnO_2_@MX sample was obtained and collected by centrifugation and washed for 3 times. For comparison, the product synthesized using the same hydrothermal process but without adding SnCl_4_⋅5H_2_O was denoted as H-MX (hydrothermal-treated MXene). 0.5-SnO_2_@MX and 2-SnO_2_@MX were also synthesized by adding half (90 mg) and double (360 mg) the amount of SnCl_4_⋅5H_2_O, respectively.

### Preparation of SnO_2_@MX-PP and MX-PP

The obtained SnO_2_@MX was thoroughly washed with N-methyl-2-pyrrolidone (NMP, Merck) with the aid of a vortex mixer and centrifuged at 15,000 rcf for 3 times. The resulting sediment was SnO_2_@MX mixed with NMP solvent. Then, the solvent exchanged SnO_2_@MX was slightly diluted with NMP and ground to form a uniform paste, which was subsequently blade coated onto one side of the PP (polypropylene) separator. The coated separator was then vacuum dried overnight at 60 °C. According to the necessity, the same process could be repeated for the modification of the other side after the first side was completely dried. Finally, the SnO_2_@MX-modified PP separator was punched into wafers with a diameter of 19 mm. The loading of SnO_2_@MX in modified PP was about 0.1 mg cm^−2^. The MX-PP was processed using the same method, but with SnO_2_@MX replaced by MXene.

More details of other syntheses and characterizations can be seen in Supporting Information.

## Results and Discussion

### Theoretical Calculation

**Fig. 1:**
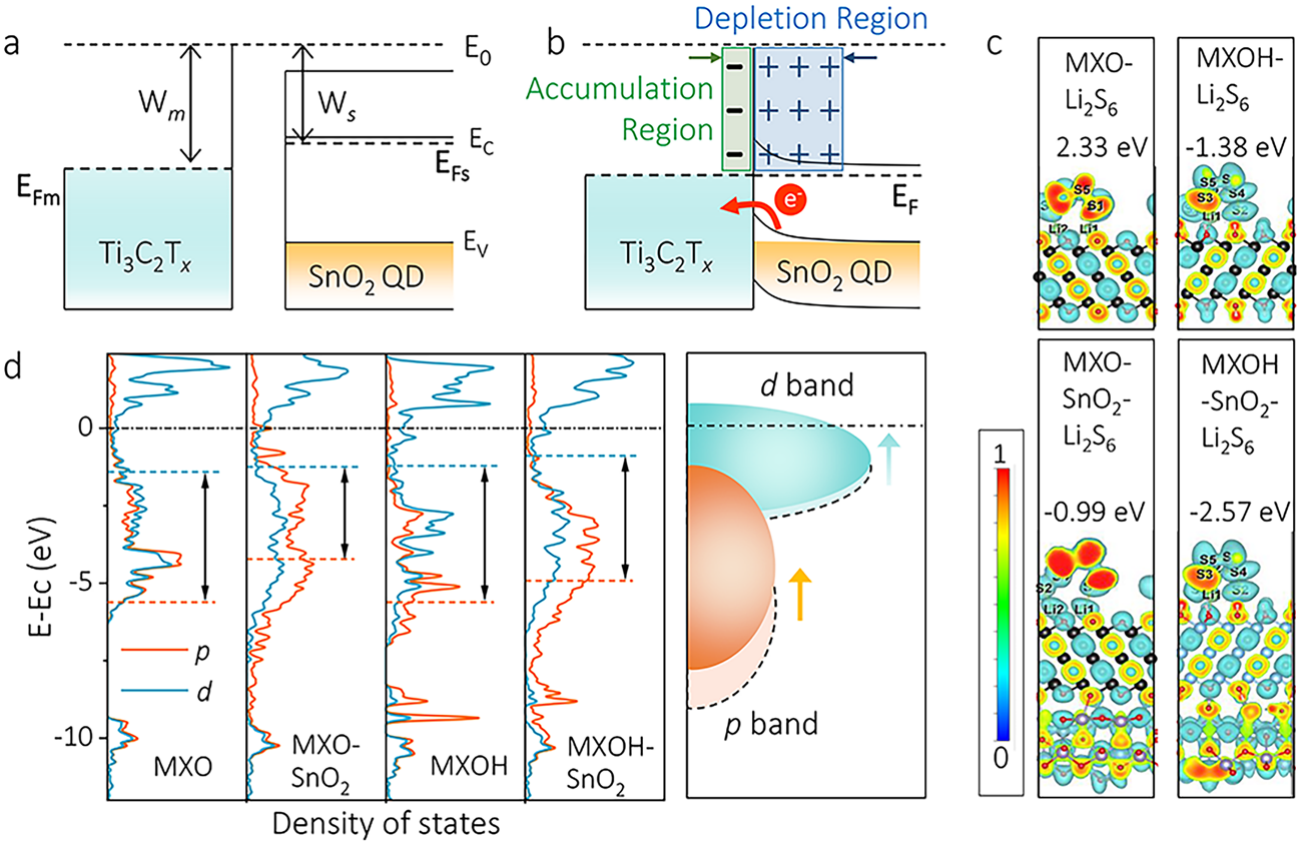
Energy band diagram of Mott–Schottky type contact between MXene (work function W_*m*_ = 4.37 eV) and SnO_2_ (work function W_*s*_ = 3.84 eV) **a** before and **b** after contacting. **c** Electron localization functions and the Li_2_S_6_ adsorption energy on the –O-terminated MXene (abbreviated MXO in the figure), SnO_2_-supported -O-terminated MXene (abbreviated MXO-SnO_2_ in the figure), –OH-terminated MXene (abbreviated MXOH in the figure), and SnO_2_-supported –OH-terminated MXene (abbreviated MXOH-SnO_2_ in the figure), from left to right. The color presents the degree of localization of electrons, e.g., blue (localization) to red (delocalization). The surface level is set to 0.6. **d** Partial density of states (PDOS) analysis of metal *d* and non-metal *p* bands in both MXO and MXOH, with or without SnO_2_ support. The energy gaps between *p*- and *d*-band center for MXO, MXO-SnO_2_, MXOH, and MXOH-SnO_2_ are 4.18, 2.92, 4.41, and 4.04 eV

To understand the roles of the Mott–Schottky heterostructure and its adsorption/catalytic mechanism in our work, we provide a schematic of the formed SnO_2_@MX heterojunction. As depicted in Fig. 1a and b, when metallic Ti_3_C_2_T_*x*_ MXene contacts semiconducting SnO_2_, electron transfer from SnO_2_ to MXene occurs to equilibrate the respective Fermi levels. Note that the work function of Ti_3_C_2_T_*x*_ MXene is higher than that of SnO_2_ according to the literature [43, 44]. This electron transfer results in the formation of a depletion region carrying a positive charge on the SnO_2_ side and an accumulation region with a negative charge on the MXene side, leading to the generation of an internal electric field, commonly known as built-in electric field (BIEF). Figure S1 shows the direction of the electric field on the surface of the SnO_2_@MX heterostructure. Significantly, the electron coordination environment in both of these regions changes from the initial state, impacting the electronic structure and influencing the capability of surface adsorption as well as the catalytic activity toward LiPSs.

Density functional theory (DFT) calculations were performed to explore the supporting role of SnO_2_ on Ti_3_C_2_T_*x*_ (T=O or OH as representative) along with the adsorption behavior toward the Li_2_S_6_ cluster. Figures 1c and S2 reveal the pronounced interfacial effect of the SnO_2_ slab on the surface charge modulation. In the presence of SnO_2_, charge localization slightly reduces, aligning with the enhanced negative adsorption energy values (Table S1). This underscores the instrumental role of SnO_2_ slab in facilitating adsorption of the Li_2_S_6_ cluster. Partial density of states (PDOS) of *d*-band and non-metal *p*-band were further analyzed to elucidate changes in the band structure brought about by the SnO_2_ slab (Fig. 1d and Table S2). Upon integration with SnO_2_, both the *d*-band and non-metal *p*-band centers upshift with respect to the Fermi level. Moreover, the energy gaps between *d*- and anion *p*-band center are reduced, which indicates reduced energy gaps between bonding and antibonding orbitals, thereby facilitating the electron transfer and consequently enhancing the conversion of LiPSs. Moreover, a further analysis of bonding and antibonding states was conducted using the crystal orbital overlap population (COOP), as shown in Fig. S3. The SnO_2_-supported MXene exhibited enhanced bonding states, indicated by a positive sign in COOP and a negative sign in crystal orbital Hamilton population (COHP), compared to its counterpart without the SnO_2_ slab. This observation aligns well with the calculated adsorption energy. Above results demonstrate that the formation of a heterostructure on the MXene plane can regulate the adsorption strength and facilitate the catalytic activity, offering significant promise in promoting the reversible transformation of LiPSs.

### Characterization of SnO_2_@MX Heterostructure

**Fig. 2:**
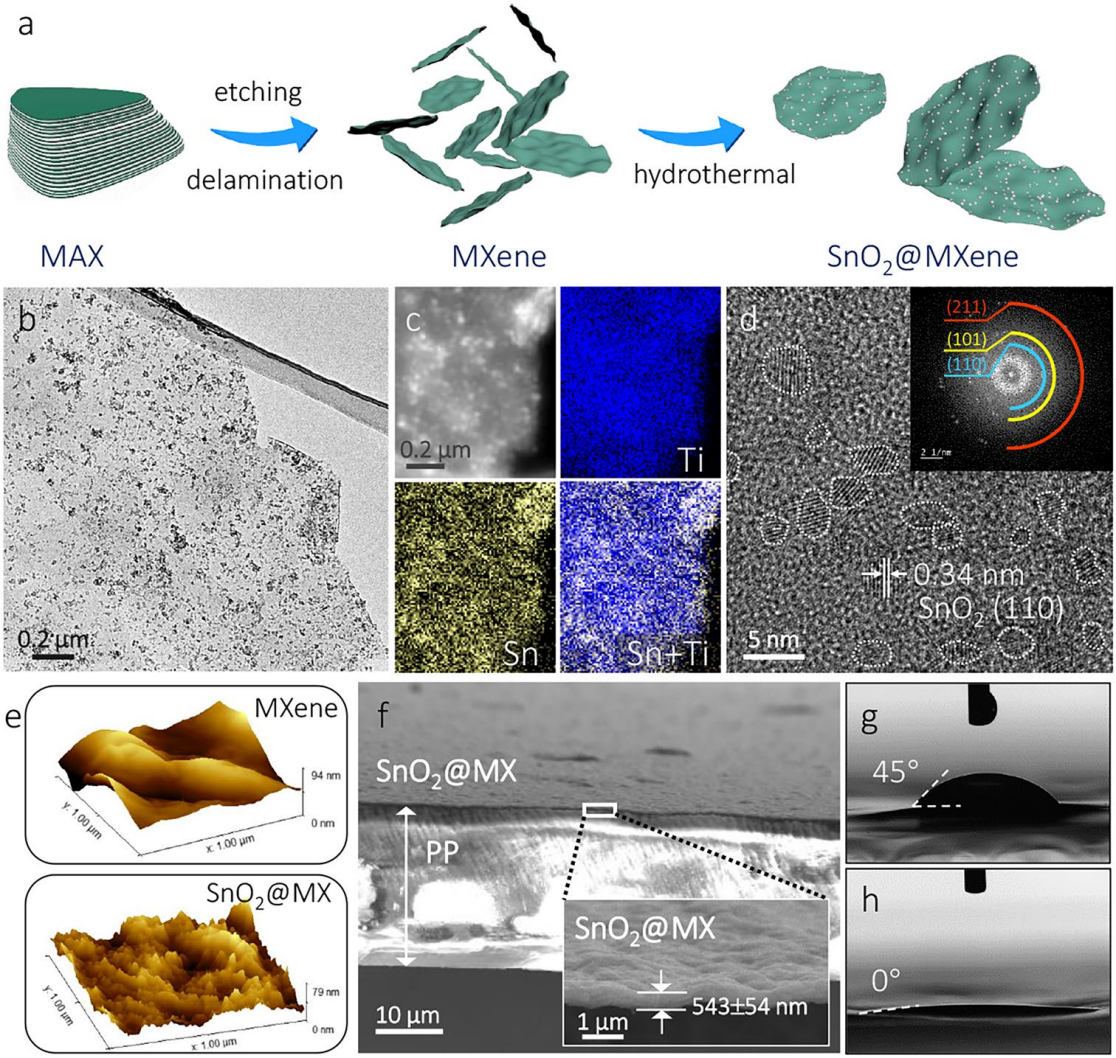
**a** Schematic diagram of the synthesis process of SnO_2_@MX. **b** TEM image of SnO_2_@MX. **c** EDS elemental mapping images of SnO_2_@MX. **d** HRTEM image with fast Fourier transform (FFT) pattern of SnO_2_@MX. **e** AFM image of MX and SnO_2_@MX interlayer surface. **f** Cross-section SEM image of SnO_2_@MX-PP separator. Contact angles between electrolyte and **g** PP and **h** SnO_2_@MX-PP separator

Motivated by above theoretical calculations, a 0D–2D Mott–Schottky SnO_2_@MX heterostructure was fabricated. As shown in Fig. 2a, the delaminated Ti_3_C_2_T_*x*_ MXene was first synthesized via the MILD route based on commercial Ti_3_AlC_2_ powder. After a hydrothermal procedure, the SnO_2_@MX heterostructure with nucleated SnO_2_ seeds decorating the MXene nanosheets (named as SnO_2_@MX) was obtained. The ultrathin MXene nanosheets appear almost transparent under the electron beam (Fig. S4), and SnO_2_ is distinctly visible and generally dispersed across the MXene flakes (Fig. 2b). We also observed instances of localized aggregation, suggesting that the dispersion, although predominantly homogeneous, includes areas where SnO_2_ particles have clustered (Fig. S5). The distribution is further confirmed by energy-dispersive X-ray spectroscopy (EDS) elemental mapping (Fig. 2c). The high-resolution transmission electron microscope (HRTEM) analysis reveals the presence of rutile SnO_2_ QDs, as evidenced by the crystal spacing and diffraction rings of (110), (101), and (211) planes (Fig. 2d). The average size of the QDs is ~ 3.9 nm (Fig. S6). Such small particles ensure numerous heterojunction sites, maximizing the utilization efficiency of the SnO_2_ material.

It is interesting to note that, compared to MXene, the structural morphology of SnO_2_@MX remains largely unchanged (Fig. S7), which enables the fabrication of a compact, low-porosity laminar structure suitable as a separator interlayer in Li–S batteries. As shown in Fig. S8, SnO_2_@MX slurries can be directly cast onto the PP separator using an industrially compatible doctor-blade technique, with no additional binders required. The as-prepared SnO_2_@MX-modified PP (SnO_2_@MX-PP) was characterized by scanning electron microscopy (SEM). Figure S9 shows MXene and SnO_2_@MX covering the porous PP separator. It is worth noting that the MXene-modified PP (MX-PP) exhibits distinct grooved stripes, which may be attributed to the different surface properties of the two materials. Atomic force microscopy (AFM) characterization reveals that the surface roughness of the SnO_2_@MX interlayer is significantly higher than that of the MXene interlayer, which suggests a substantial number of nanoparticles decorating the MXene plane (Fig. 2e). In fact, SnO_2_@MX shows better adhesion to the PP separator (compared to MXene) with remarkable structural and mechanical robustness (Fig. S10). The cross-sectional SEM image reveals the dense parallel stacking of SnO_2_@MX nanosheets, with a thickness of 543 ± 54 nm (Fig. 2f). Such ultrathin thickness and negligible mass effectively minimize its impact on the cell energy density. Additionally, the wettability of the separator interlayer was also validated using contact angle (CA) measurements. In contrast with the relatively stable CA (≈ 45°) formed between PP separator and electrolyte, the electrolyte rapidly spreads on both SnO_2_@MX-PP and MX-PP, resulting in a CA close to 0° (Figs. 2g and S11). This indicates excellent wettability of both SnO_2_@MX and MXene separators by the electrolyte, which guarantees efficient interactions with LiPSs and is beneficial for Li ion transportation.

**Fig. 3:**
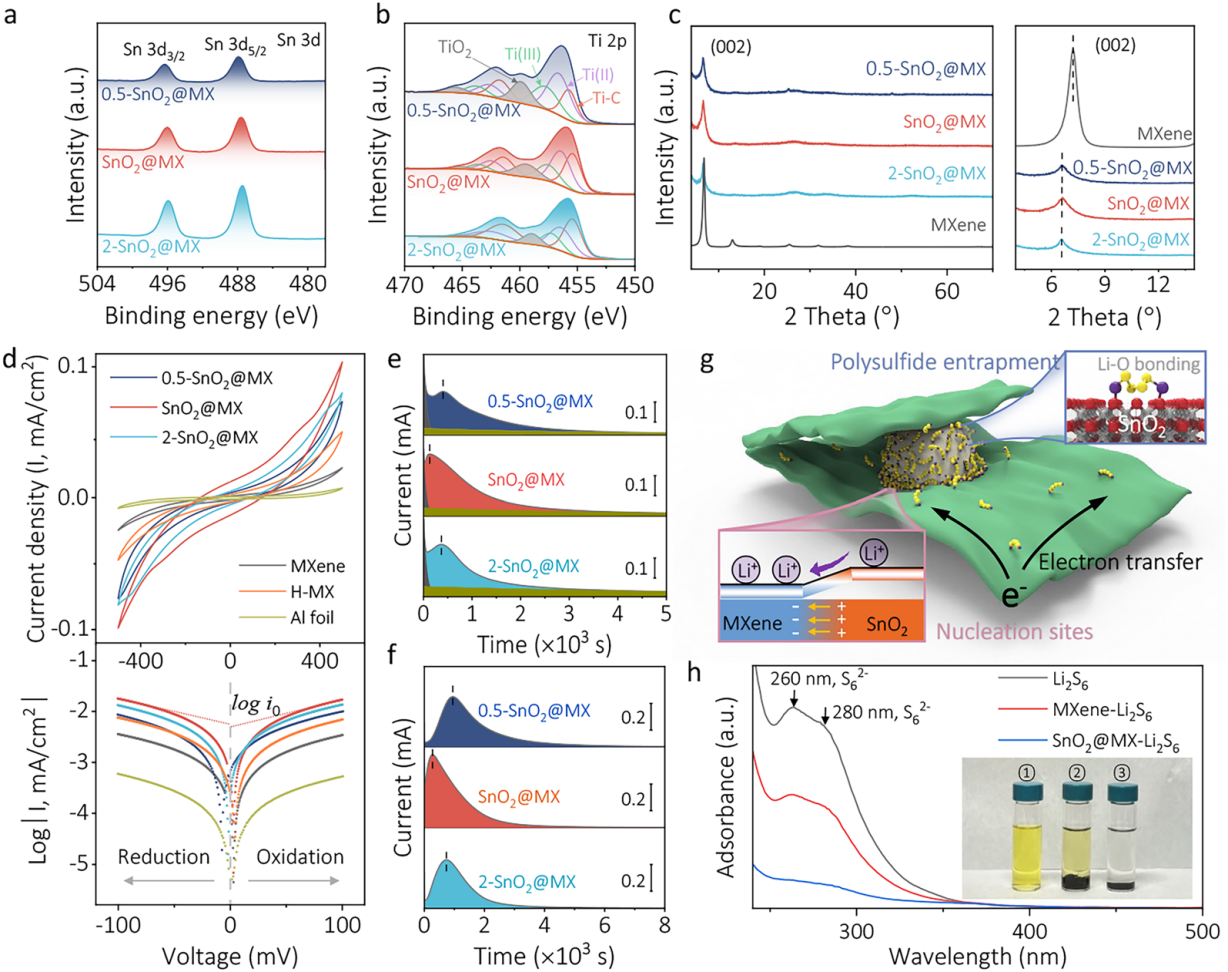
XPS analyses of **a** Sn 3*d* and **b** Ti 2*p* in 0.5-SnO_2_@MX, SnO_2_@MX, and 2-SnO_2_@MX (these three samples are collectively termed SnO_2_@MXs in the following). **c** XRD patterns of MXene and SnO_2_@MXs. Electrocatalytic activity tests of **d** CV curves (up) of symmetric cells with Li_2_S_6_ dissolved in electrolyte as active material and the corresponding Tafel plots (down) with the scan rate of 10 mV s^−1^. Potentiostatic **e** precipitation and **f** dissolution tests of SnO_2_@MXs electrodes with Li_2_S_8_ dissolved in the electrolyte. **g** Schematic illustration of the efficient LiPSs adsorption and catalytic conversion process in SnO_2_@MX heterostrctures, where the 2D flakes are MXene (green color), LiPSs chains are adsorbed on SnO_2_ (sphere with white color) surface. **h** Adsorption test using UV–Vis spectra of Li_2_S_6_ in DOL and after adsorption by MXene and SnO_2_@MX powder, with inset showing a digital image of the solution from above after equilibration for 24 h. The solution from left to right numbered one to three, is, respectively, Li_2_S_6_, MXene-Li_2_S_6_, and SnO_2_@MX-Li_2_S_6_ solution

To explore the chemical states of SnO_2_@MX, including variations with different Sn concentrations, we synthesized the SnO_2_@MX samples with varying amount of Sn precursors: SnO_2_@MX, 0.5-SnO_2_@MX, and 2-SnO_2_@MX (refer to the experimental section for details). Additionally, a synthesis omitting Sn precursor was conducted, resulting in a variant denoted as H-MX to further our comparative analysis. X-ray photoelectron spectroscopy (XPS) analysis was performed to gain insight into the valance states of Sn and Ti. In the Sn 3*d* spectra, doublet peaks at 487.4 and 495.8 eV correspond to the 3*d*_5/2_ and 3*d*_3/2_ orbitals of SnO_2_, respectively (Fig. 3a). The peak intensities increase with more Sn precursor used. The Ti 2*p* spectra for Ti_3_C_2_T_*x*_ exhibit doublets at 455.6/461.6, 456.4/462.4, and 457.6/463.6 eV, with a 6.0-eV splitting energy. And the doublet at 459.6/465.3 eV is attributed to TiO_2_. Even though the TiO_2_ peaks in the SnO_2_@MX samples are higher than in pristine MXene, they are noticeably smaller than the TiO_2_ peaks emerging in H-MX, indicative of MXene oxidation during the hydrothermal process (Fig. S12). In the SnO_2_@MX samples, the higher Sn precursor concentration, the smaller the TiO_2_ peak intensity. This can be ascribed to the tight decoration of the SnO_2_ seeds, which isolates MXene flakes from direct exposure to aqueous solution [45]. The TEM image shows TiO_2_ nanocrystal rods (~ 50 nm in length) in H-MX (Fig. S13). Nevertheless, there are no distinct TiO_2_ observed in the XRD of H-MX and SnO_2_@MX samples, which could be attributed to the limited oxide content (Figs. 3c and S14). In MXene, we do observe the characteristic peak of MXene at 2*θ* = 7.2° for (002). Interestingly, both H-MX and SnO_2_@MX samples exhibit a shift of this peak toward lower angles, with 2θ values of 6.3° and 6.6°, respectively. According to Bragg’s law, this shift toward lower 2θ angles implies an increase in the *d*-spacing, indicating expanded interlayer spacing (Figs. S15 and S16). Such expansion is beneficial for Li^+^ transportation in Li–S batteries, as will be discussed below.

### Electrocatalytic Optimization of SnO_2_@MXs and Adsorption Behavior Toward LiPSs

To optimize the SnO_2_ content in SnO_2_@MX for maximum catalytic activity and investigate the catalytic effect of TiO_2_ derived from MXene, kinetics evaluations were further conducted on the prepared samples. Li_2_S_6_ symmetric cells were assembled to assess the transformation kinetics of liquid-phase LiPSs. The CV curves in Fig. 3d indicate that SnO_2_@MX displays the highest current response, suggesting its superior catalytic ability for LiPSs conversion. The exchange current density (*i*_0_) can be determined by fitting the experimental curve to the Tafel equation, with SnO_2_@MX distinctly showcasing superior conversion kinetics, as evidenced by its highest |log *i*_0_| values. Potentiostatic experiments were performed to analyze the nucleation and dissolution processes of Li_2_S. Figures 3e and S17 show the potentiostatic precipitation curves of Li_2_S, where the current peak stems from the nucleation of Li_2_S followed by growth to impingement [46]. The results demonstrate the highest Li_2_S nucleation and growth rate of SnO_2_@MX with the earliest peak response time (t_m_ = 135 s) and highest Li_2_S deposition capacity (273 mAs). The potentiostatic dissociation curves of Li_2_S, shown in Figs. 3f and S18, depict the decomposition process of solid Li_2_S. Notably, SnO_2_@MX continues to demonstrate the fastest peak response time and dissolution capacity. These results indicate that SnO_2_@MX exhibits optimal catalytic activity not only for the interconversion of LiPSs but also for the redox process of solid Li_2_S, highlighting the high efficiency of SnO_2_ over oxidized TiO_2_. Figure 3g illustrates the role of SnO_2_@MX catalyst in Li–S cells. The well-constructed heterostructure enriches the electrochemical active sites at phase boundaries, lowering Li_2_S nucleation barriers, boosting charge transfer and conversion kinetics, and ensuring the re-utilization of LiPSs.

To investigate the LiPSs adsorption behavior, an adsorption test was performed using a Li_2_S_6_ solution. As shown in Fig. 3h, the Li_2_S_6_ solution immersed with SnO_2_@MX turned colorless after standing still for 24 h, whereas the LiPS solution with an equal amount of MXene still exhibited a light yellow color. UV–Vis spectra further confirmed the absence of LiPS in the supernatant after immersion, as evidenced by the weakened peaks at 260 and 280 nm. The SnO_2_@MX-Li_2_S_6_ group demonstrated the lowest peak intensity as well as the lightest solution color, indicating the strongest adsorption capability of SnO_2_@MX toward LiPSs. To visualize the inhibition of LiPSs migration through the separator, diffusion tests were performed using a H-typed glass apparatus (Fig. S19). It was observed that both the PP and MX-PP separators showed obvious LiPSs penetration after 24 h. However, the migration of LiPSs across the SnO_2_@MX-PP separator was effectively restrained. Therefore, the SnO_2_@MX-PP separator demonstrates superior inhibition of the diffusion of LiPSs, thus contributing to significant suppression of the shuttle effect in Li–S batteries.

### Comparison of Electrochemical Performance

**Fig. 4:**
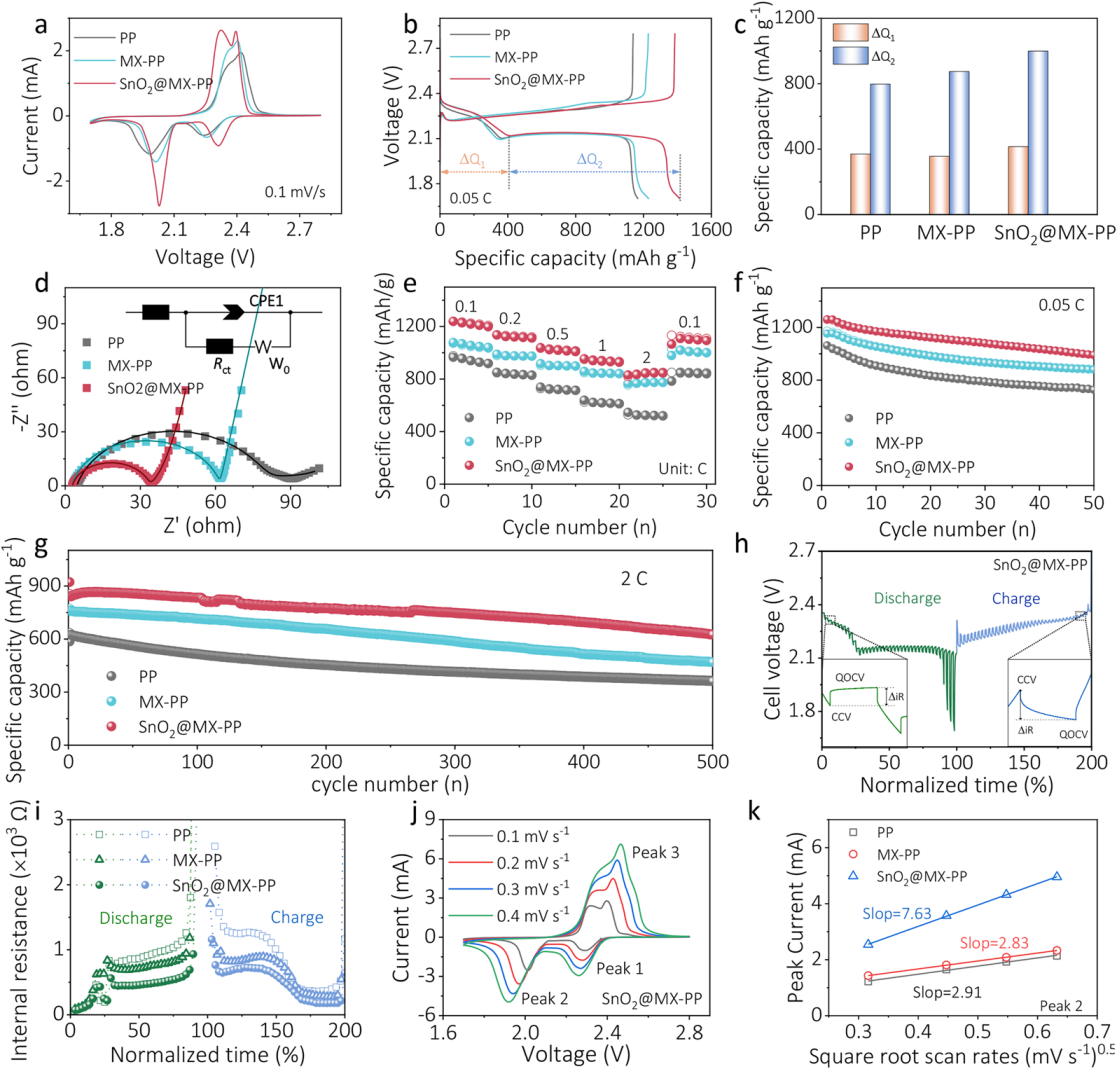
**a** CV curves of the Li–S coin cells with various separators (PP, MX-PP, and SnO_2_@MX-PP) at a scan rate of 0.1 mV s^−1^. **b** Charge/discharge profiles of cells with various separators and **c** the corresponding comparison of the first discharge plateau (ΔQ_1_) and the second discharge plateau (ΔQ_2_) capacities. **d** Nyquist plots of Li–S cells with various separators from EIS tests before cycling. **e** Rate performance and cyclic performances at **f** 0.05 C and **g** 1 C of Li–S cells with various separators (In the graph, solid markers represent discharge, and hollow markers represent charging. The cells were tested after 1 cycle of activation process). **h** GITT plots of Li–S cell with SnO_2_@MX-PP separator and **i** the derived internal resistance plots of cells with the three separators with respect to normalized time. **j** CV curves of the cell with SnO_2_@MX-PP separator at gradient scan rates and **k** the plots of the peak 2 current versus the square root of scan rates

To evaluate the potential application of the optimized SnO_2_@MX interlayer in Li–S batteries, the electrochemical behaviors of a series of coin cells were tested with CNT (carbon nanotubes)/S as cathode and SnO_2_@MX-modified PP as separator (Fig. S20). Figure 4a shows the cyclic voltammetry (CV) curves of cells with PP, MX-PP, and SnO_2_@MX-PP separators at a scan rate of 0.1 mV s^−1^, respectively. Two distinct cathodic peaks located at 2.2 ~ 2.3 and 1.9 ~ 2.0 V correspond to the liquid–liquid phase transformation and liquid–solid phase transition of sulfur species. Conversely, the oxidation process exhibits overlapping peaks, resulting in an anodic peak located at 2.3 ~ 2.4 V. Notably, the cell with SnO_2_@MX-PP separator demonstrates significantly higher peak current and lower voltage polarization, indicative of improved conversion kinetics in the Li–S cell. The galvanostatic charge–discharge (GCD) profiles at 0.05 °C are displayed in Figs. 4b and S21. The two discharge plateaus and one charge plateau in the curves are consistent with the CV peaks. Remarkably, the cell with SnO_2_@MX-PP separator demonstrates a significantly higher initial discharge capacity of 1414 mAh g^−1^, surpassing both PP- and MX-PP-based Li–S cells. The specific capacities for the first and second plateau, denoted as ΔQ_1_ and ΔQ_2_, respectively, are illustrated in Fig. 4c. We find SnO_2_@MX-PP displays a capacity boost in both plateaus, with more pronounced increase in the second plateau (25.3%) than the first (12.1%). Electrochemical impedance spectroscopy (EIS) measurements show a smaller charge-transfer impedance (*R*_ct_) for SnO_2_@MX-PP compared to MX-PP and PP cells (Fig. 4d and Table S3), indicating superior redox kinetics in the SnO_2_@MX-PP system.

Rate capabilities of cells were compared under a stepwise increase in current rates from 0.1 to 2 °C (Fig. 4e). The cell with SnO_2_@MX-PP separator consistently exhibits superior capacity at each gradient of current rate. Even at a high current rate of 2 °C, a satisfactory capacity of 845 mAh g^−1^ is obtained. After cycling at varying rates and returning to 0.1 °C, the capacity recovers to 1193 mAh g^−1^ (30th cycle), nearly matching the initial 1198 mAh g^−1^ (5th cycle). Figure 4f shows the cyclic performance evaluated at a small current rate of 0.05 °C. After activation, the SnO_2_@MX-PP outperforms both MX-PP and PP, maintaining 991 mAh g^−1^ after 50 cycles, equivalent to 79% of capacity retention, compared to 76% for MX-PP and 68% for PP, respectively. This underscores the SnO_2_@MX interlayer’s efficiency in enhancing sulfur utilization and reducing capacity decay by suppressing LiPSs shuttling and promoting their conversion. The long-term cyclic performance was also studied under a high current rate of 2 °C (Figs. 4g and S22). The SnO_2_@MX-PP delivers an initial discharge capacity of 844 mAh g^−1^, which reduces to 625 mAh g^−1^ after 500 cycles (0.052% decay per cycle), while MX-PP and PP start at 760 and 624 mAh g^−1^, decaying at 0.069% and 0.084% per cycle, respectively. This highlights the superior capacity and enhanced long-term stability of the SnO_2_@MX-PP in Li–S batteries. After undergoing and extensive aging within the cell, the SnO_2_@MX-PP is extracted and further analyzed, demonstrating a decent stability of SnO_2_@MX composite (Fig. S23).

Galvanostatic intermittent titration technique (GITT) measurements were applied to examine the internal resistances of the cells employing different separators (Figs. 4h and S24). The polarization occurring during electrochemical operation is quantified by determining the internal resistance ($\Delta {R}_{\text{inter}}$) using the following equation:

1 $$ \Delta R_{{{\text{inter}}}} \left( \Omega \right) = \left| {\Delta V_{{{\text{QOCV}} - {\text{CCV}}}} } \right|/I_{{{\text{appli}}}} $$

where $\Delta {V}_{\text{QOCV}-\text{CCV}}$ represents the voltage difference between the quasi open-circuit voltage (QOCV) and closed-circuit voltage (CCV), and ${I}_{\text{appli}}$ is the applied current. The values of $\Delta {R}_{\text{inter}}$ are plotted in Fig. 4i for the three cells based on different separators, as function of normalized time for both charge and discharge processes. It is interesting to find that the $\Delta {R}_{\text{inter}}$ in Li_2_S formation and dissolution regimes is significantly higher than in LiPSs conversion regimes, indicating Li_2_S-related reactions encounter the highest energy barrier during charge and discharge. Moreover, the SnO_2_@MX-PP demonstrates smaller $\Delta {R}_{inter}$ values throughout the entire process, suggesting lower internal resistance and enhanced reaction kinetics than the other cells. Li^+^ diffusivity was analyzed using sequential CV measurement on Li–S cells at various scan rates (Figs. 4j and S25), identifying two cathodic and one anodic peak as peaks 1, 2, and 3. The relationship between the peak current (${I}_{p}$) and scan rate ($\upnu$) can be described by the Randles–Sevcik equation [47]:

2 $$ I_{p} = 2.69 \times 10^{5} Az^{1.5} D_{Li}^{0.5} c\nu^{0.5} $$

where ${D}_{Li}$ is Li^+^ diffusion coefficient, $z$ is the number of transferred charges, $A$ is the surface area of the electrode, and $c$ is the Li^+^ concentration. The peak current (${I}_{p}$) exhibits a linear relationship with ${\nu }^{0.5}$, where the slope is associated with ${D}_{Li}^{0.5}$. As shown in Figs. 4k and S17, the SnO_2_@MX-PP displays a higher slope compared to MX-PP and PP, with calculated Li^+^ diffusion coefficients of 2.97 × 10^–7^, 4.70 × 10^–7^, and 1.52 × 10^–6^ cm^2^ s^−1^ for peaks 1, 2, and 3, respectively. These results suggest that incorporating the SnO_2_@MX-PP interlayer effectively boosts the Li^+^ diffusion rate and promotes the kinetics of the LiPSs redox process.

### Lithium Dendrite Growth Suppression by SnO_2_@MX

**Fig. 5:**
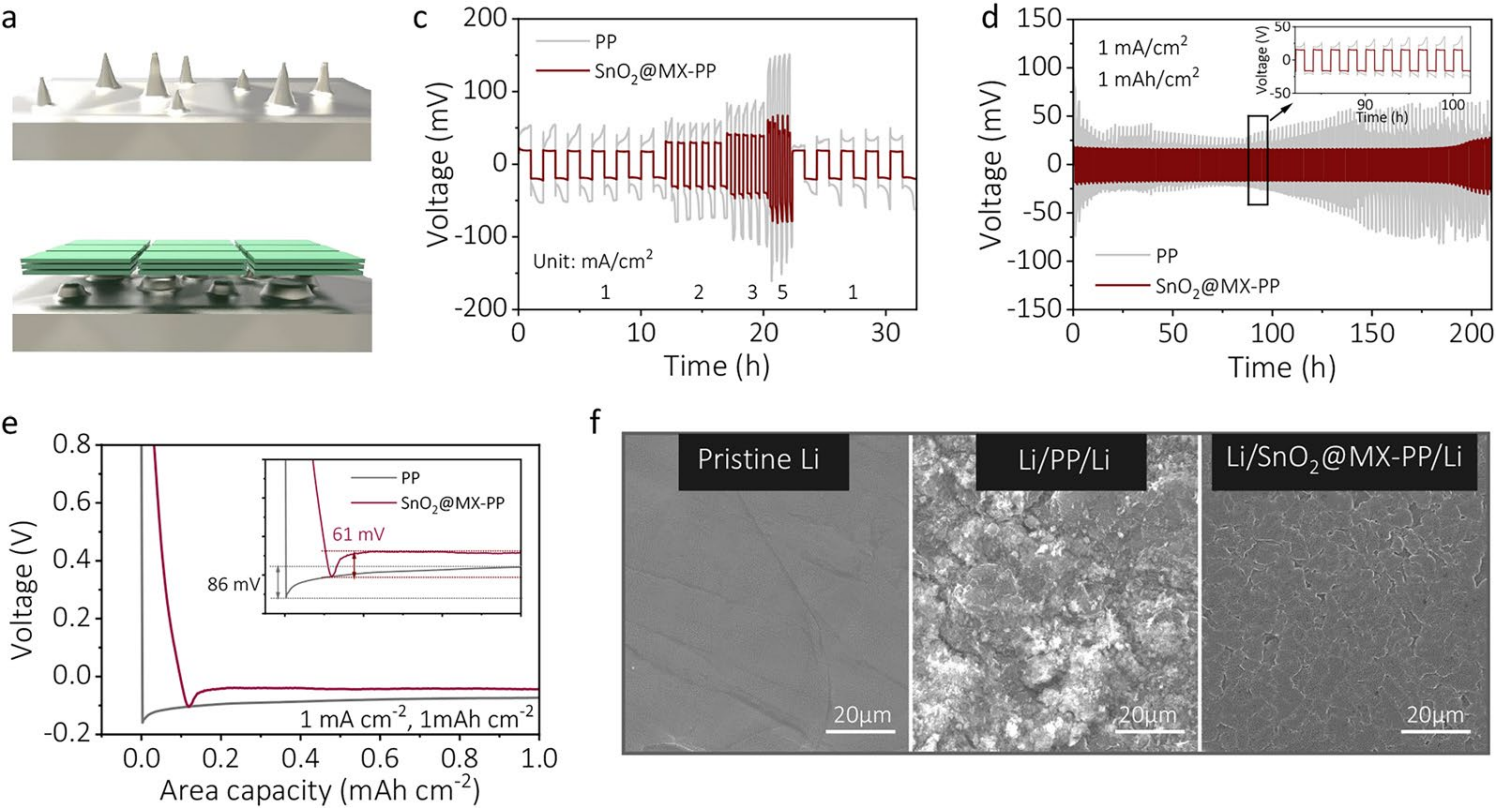
**a, b** Schematic diagram of inhibition of lithium dendrite growth. **c** Rate performance and **d** cyclic performance of Li/Li symmetric cells with PP or SnO_2_@MX-PP separator. **e** Voltage–capacity profile of lithium plating and stripping in Li/Cu cell. **f** SEM of pristine Li and Li electrode surface with various separators after 400 cycles

Since Li dendrite growth in the Li anode poses safety risks to pierce the separator and cause short circuits, the introduction of SnO_2_@MX is believed to be beneficial in suppressing Li dendrite formation due to the high Young’s modulus, efficient Li^+^ conduction ability, and lithophilic sites (Fig. 5a and b) [48]. To validate this, symmetric Li/Li cells were assembled and subjected to galvanostatic cycling to assess the lithium plating/stripping behaviors. Figure 5c shows the voltage profiles of the symmetric cells at different current densities of 1, 2, 3, and 5 mA cm^−2^. Notably, the SnO_2_@MX-PP Li/Li cell exhibits superior rate performance with consistently lower overpotential than the PP-based cell. During long-term cycling at 1 mA cm^−2^ (Fig. 5d), the SnO_2_@MX symmetric cell exhibits superior stability over 200 h, with consistent voltage hysteresis and only about 20-mV polarization. Meanwhile, the PP-based cell displays fluctuating overpotentials (> 20 mV), indicating significant polarization and unstable solid electrolyte interface (SEI) formation. Li/Cu cells were also constructed to assess nucleation overpotentials. As shown in Fig. 5e, the SnO_2_@MX electrode exhibits a reduced nucleation overpotential (61 mV) compared to that of bare Cu electrode (86 mV), underscoring its capability to guide uniform Li plating by reversing dendrite growth. This is further confirmed by the SEM images in Figs. 5f and S26, as a smooth, flat surface is found in cycled Li electrode from the SnO_2_@MX-PP-based cell while a rough and patchy surface is observed in the PP-based cell. Thus, the SnO_2_@MX interlayer not only exerts an advantageous effect on the cathode but also demonstrates the ability of inhibiting Li dendrite formation on the anode side.

### Evaluation of High Sulfur Loading Performance

**Fig. 6:**
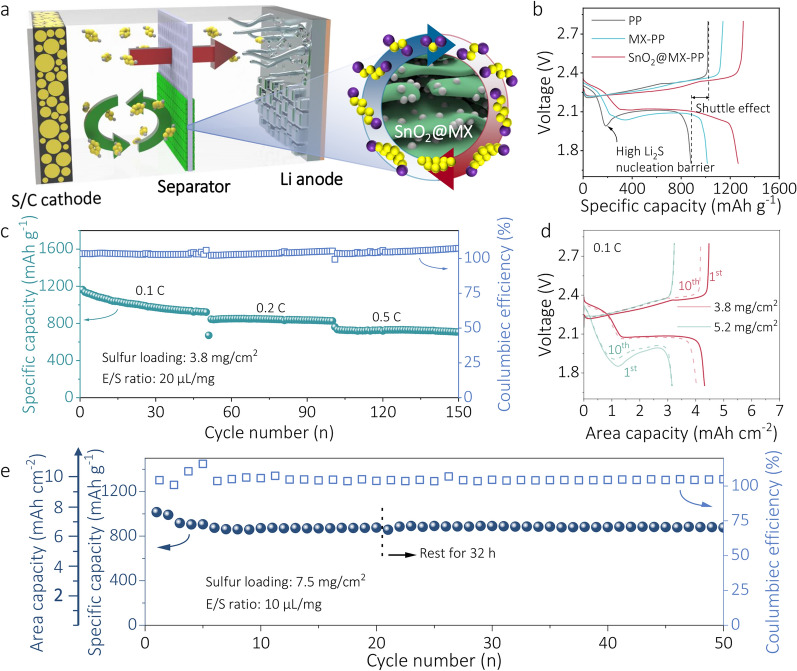
**a** Schematic diagram of the function of SnO_2_@MX coating on both cathode and anode sides. Electrochemical performances of **b** charge–discharge curves with PP, MX-PP, and SnO_2_@MX-PP separator at 0.05 °C with sulfur loading of 3.8–4.0 mg cm^−2^ and E/S (electrolyte/sulfur) ratio of 20 μL mg^−1^. **c** Cyclic performance of SnO_2_@MX-PP at different rates with a sulfur loading of 3.8 mg cm^−2^ and E/S ratio of 20 μL mg^−1^. **d** Charge–discharge curves of the first (solid lines) and 10th (dot lines) cycle with CNT various sulfur loadings at 0.1 °C. **e** Cyclic performance of SnO_2_@MX-PP at 0.02 °C with a sulfur loading of 7.5 mg cm^−2^ and E/S ratio of 10 μL mg^−1^

Considering the practical application of Li–S batteries, the performance of cells with high sulfur loading and SnO_2_@MX-PP separator was further investigated. The SnO_2_@MX heterostructure interlayer was coated on both sides of the PP separator, providing functions to suppress shuttling and catalyze the conversion of LiPSs, as well as inhibiting Li dendrite growth on the other side (Fig. 6a). Figure 6b shows the initial charge–discharge profiles of SnO_2_@MX-PP-, MX-PP-, and PP-based Li–S cells under a high sulfur loading of 3.8–4.0 mg cm^−2^. A voltage valley before the second discharge plateau in the PP-based cell indicates a high-energy barrier for Li_2_S nucleation. The overcharging observed during the charging process also suggests a severe shuttle effect under high sulfur loading conditions [49]. In contrast, the SnO_2_@MX-PP-based cell exhibits facilitated Li_2_S nucleation and inhibition of LiPSs shuttling. The presence of highly catalytic heterojunction sites significantly lowers the nucleation energy, enabling more efficient nucleation without noticeable barrier. Figure 6c displays the cyclic performance of SnO_2_@MX-PP at various rates. The cell achieves an initial capacity of ~ 1160 mAh g^−1^ at 0.1 °C and maintained stability over 50 cycles, even at higher current rates of 0.2 and 0.5 °C. However, increasing the sulfur loading from 3.8 to 5.2 mg cm^−2^ led to higher overpotential and reduced area capacity from ~ 4 to 3 mAh cm^−2^ (Fig. 6d). Nevertheless, a decent capacity of 4.7 mAh cm^−2^ can still be achieved at lower current rate of 0.05 °C in a high sulfur loading of 5.7 mg cm^−2^ (Fig. S27). Furthermore, when the sulfur loading was increased up to 7.5 mg cm^−2^, the cell realized an initial area capacity of over 7.6 mAh cm^−2^ and maintained stability at a 0.02 °C current rate (Fig. 6e). This higher stability over 50 cycles compared to that shown in Fig. 4f could be attributed to the lower depth of discharge levels. Nevertheless, challenges persist, including uneven electrolyte wetting and increased polarization at lower E/S ratios (Fig. S28). Table S4 presents the comparison of electrochemical performance with other recent reported works. All in all, the SnO_2_@MX-modified PP separator effectively reduces Li_2_S nucleation overpotential, inhibits Li dendrite growth as well as the shuttle effect, thereby enabling decent performance with high sulfur loadings. However, achieving high capacity with low overpotentials remains challenging when considering both high sulfur loading and current rates. Separator modification alone may not fully meet the requirement for commercialization, and it is more promising to combine novel separators like the ones developed in this work with other advancements in cathode, electrolyte, and anode technologies. Still, developing an ultrathin and efficient separator interlayer remains crucial.

## Conclusion

In summary, we have developed a dense, ultrathin, and laminar SnO_2_@MX heterostructure separator interlayer to effectively suppress LiPSs shuttling, catalyze redox conversions, and inhibit Li dendrite growth in Li–S batteries. Specifically, SnO_2_ QDs were integrated onto the MXene basal plane, creating boundary sites with coordination environments that enhance LiPSs immobilization and rapid charge transfer. The synergistic effects of SnO_2_, MXene, and their heterojunctions effectively modulate the reaction kinetics, encompassing LiPSs trapping, diffusion to boundaries, Li_2_S nucleation, growth, and dissolution. Thanks to the unique properties of the heterostructure and the structural advantage of the SnO_2_@MX interlayer, the SnO_2_@MX-PP Li–S cell exhibits superior electrochemical performances. It demonstrates a high area capacity of 7.6 mAh cm^−2^ at a high sulfur loading of 7.5 mg cm^−2^, exceptional rate capability with a capacity of 845 mAh g^−1^ at 2 °C, and remarkably cyclic stability with a capacity fading of only 0.052% per cycle over 500 cycles. Our work not only demonstrates a feasible strategy of utilizing a laminar separator interlayer for advanced commercialized Li–S batteries, but also provides valuable insights into the understanding of heterostructure catalysis and its role in boosting catalytic reaction kinetics.

## Supplementary Information

Below is the link to the electronic supplementary material.

Supplementary file1 (DOCX 5344 KB)
