# Supplementary material for: Multifunctional SnO2 QDs/MXene Heterostructures as Laminar Interlayers for Improved Polysulfide Conversion and Lithium Plating Behavior
Source: Nanomicro Lett. 2024 Jun 28;16:229. doi: 10.1007/s40820-024-01446-w (PMC11213846; doi:10.1007/s40820-024-01446-w)
Supplement: Supplementary file 1 — Supplementary file1 (DOCX 5344 KB) [file 40820_2024_1446_MOESM1_ESM.docx]

Supporting Information for

**Multifunctional SnO_2_ QDs/MXene Heterostructures as Laminar Interlayers for Improved Polysulfide Conversion and Lithium Plating Behavior**

Shungui Deng^1,2,3^, Weiwei Sun^4,5^, Jiawei Tang^5^, Mohammad Jafarpour^2,3^, Frank Nüesch^2,3^, Jakob Heier^2,^*, and Chuanfang (John) Zhang^1,^*

^1^ College of Materials Science & Engineering, Sichuan University, 610065 Chengdu, P. R. China

^2^ Laboratory for Functional Polymers, Swiss Federal Laboratories for Materials Science and Technology (EMPA), Überlandstrasse 129, CH-8600 Zürich, Switzerland

^3^ Institute of Materials Science and Engineering, Ecole Polytechnique Fédérale de Lausanne (EPFL), Station 12, CH-1015 Lausanne, Switzerland

^4^ Key Laboratory of Quantum Materials and Devices of Ministry of Education, School of Physics, Southeast University, Nanjing 211189, P. R. China

^5^ SEU-FEI Nano-Pico Center, Key Laboratory of MEMS of Ministry of Education, Southeast University, Nanjing, 210096, P. R. China

*Corresponding authors. E-mail: [chuanfang.zhang@scu.edu.cn](mailto:chuanfang.zhang@scu.edu.cn) (Chuanfang (John) Zhang), [Jakob.heier@empa.ch](mailto:Jakob.heier@empa.ch) (Jakob Heier)

## S1 Experimental Section

**S1.1 Characterizations**

A FEI Quanta 650 field emission scanning electron microscope (FESEM, NanoSEM 230) was used to observe the morphologies and sizes of the samples. Transmission Electron microscopy (TEM, JEOL 2200 FS, 200 kV) with an energy dispersive X-ray spectrometer (EDS) was employed to detect the detailed structure and elemental maps. Atomic force microscopy (AFM, Nanoscope Icon 3, Bruker, Germany) techniques were performed to detect the roughness of the interlayer coatings on a glass substrate. X-ray diffraction (XRD, BRUKER D8) and X-ray photoelectron spectroscopy (XPS, PHI Quantum 2000) were conducted to characterize the crystal structures and composition. Thermogravimetric analysis (TGA) was carried out using a Perkin Elmer TGA7 with heating from room temperature to 500 ℃ under a nitrogen flow at 10 ℃ min^-1^. Ultraviolet-visible (UV-vis) spectra were measured on a CARY 50 SCAN UV-vis spectrophotometer.

**S1.2 Li-S coin cell assembly and electrochemical measurements**

CNT/S was synthesized by the traditional melt-diffusion method, involving the mixing and heating of CNT powder and sublimated sulfur (S_8_, Sigma Aldrich) at 155 ℃. The resulting CNT/S composite was then combined with LA132 binder and super P, and blade-coated onto aluminum foil, followed by drying in a vacuum oven to fabricate the CNT/S cathode. CR-2032 type coin cells were assembled in an argon-filled glove box, incorporating CNT/S cathode, SnO_2_@MX-PP separator, lithium anode, and electrolyte (1:1 vol. % of DOL/DME with 1M of LiTFSI and 1 wt. % of LiNO_3_ additive). As contrast groups, SnO_2_@MX-PP was replaced by MX-PP or bare PP. For electrochemical measurements, Galvanostatic charge/discharge (GCD) tests were carried out using a multichannel battery testing system (LAND, CT-2001A, Wuhan Rambo Testing Equipment Co, Ltd.). Galvanostatic intermittent titration technique (GITT) measurements were conducted on the same system with a current pulse of 0.02 C for 1h and a rest period of 2 h. Cyclic voltammetry (CV) and Potentiostatic electrochemical impedance spectroscopy (PEIS) tests were recorded on a VSP potentiostat (BioLogic, France). In EIS, the frequency range was set from 100 kHz to 100 mHz, with an amplitude of 10 mV at open circuit voltage potential. The sulfur loading and E/S ratio for regular cells were 0.5-1.5 mg/cm^2^ and 30 μL/mg, respectively, and all tests were conducted at room temperature.

**S1.3 Visualized adsorption and diffusion tests**

For adsorption tests, a Li_2_S_6_ solution with a concentration of 2.4 mM was prepared by mixing Li_2_S and sublimed sulfur powder in a molar ratio of 1: 5 and dissolving them in the electrolyte. Then, 30 mg of freeze-dried MXene and SnO_2_@MX powder were immersed in the Li_2_S_6_ solution and left to stand still for 24 h. The whole operation procedure was conducted inside an Ar-filled glove box. For the diffusion test, 20 mM of Li_2_S_6_ was prepared and dissolved in DOL solution. A SnO_2_@MX-PP separator was inserted between two chambers, containing Li_2_S_6_ solution in one side and pure DOL solvent in other sides. The chambers was hermetically sealed and left to stand still for 24 h.

**S1.4 Assembly of Li_2_S_6_ symmetric cells**

SnO_2_@MX electrodes were fabricated by blade coating SnO_2_@MX slurry onto Al foil (carbon coated) and then cutting it into wafers. Symmetrical cells were assembled using two identical SnO_2_@MX electrodes as both cathode and anode. 20 μL 0.2 M of Li_2_S_6_ was dissolved in the electrolyte as active material. The preparation process for other electrodes and cells follows the same procedure. CV measurements were carried out in the potential range of -500~500 mV at a scan rate of 10 mV/s. The exchange current density $i_{0}$ was evaluated from the constructed Tafel plot according to the simplified Butler-Volmer equation: $\log i=\log i_{0}+\left( {\alpha F}/{2.303RT} \right)\eta$, where $i$, $\alpha$, $F$, $R$, $T$, $\eta$ are current density, charge transfer coefficient, Faraday constant, absolute temperature and overpotential, respectively.

**S1.5 Li_2_S nucleation and dissociation tests**

A Regular cell was assembled using a SnO_2_@MX electrode as cathode and Li foil as anode. 20 μL 0.2 M of Li_2_S_8_ was dissolved in the electrolyte as active material dropped onto the cathode side, and another 20 μL of electrolyte was dropped onto the anode side. For nucleation tests, the cell was first galvanostatically discharged to 2.14 V under a current of 0.112 mA, and then potentiostatically discharged at 2.12 V. For dissociation tests, the cell was initially galvanostatically discharged to 1.7 V at 0.112 mA, after 2 h of rest, the cell underwent further galvanostatically discharge to 1.7 V at 0.011 mA for a deeper discharge. Then, the cell was potentiostatically charged at 2.3 V. The process for other electrodes followed the same procedure.

**S1.6 Li plating/stripping tests**

In this test, Li//Li and Li//Cu cells were assembled. For Li//Li cell assembly, Li metal with a diameter of 15 mm was used for both cathode and anode. The separator options include PP or SnO_2_@MX-PP (SnO_2_@MX interlayer coated on both side) as comparison group. For Li//Cu cell assembly, Li metal was applied as anode, and a 13 mm diameter Cu foil served as cathode, with PP or SnO_2_@MX-PP (SnO_2_@MX interlayer coated on the cathode side) as separator. The effective area of Cu foil was considered as the geometric surface area in Li//Cu cells. The Li plating or stripping process was conducted under a current density of 1 mA/cm^2^ and a capacity of 1 mAh/cm^2^. The usage of electrolyte for both cells was 75 μL.

**S1.7 Computational method**

Density functional theory (DFT) calculations were performed using the Vienna Ab-initio Simulation Package (VASP) [1]. For the ion-electron interactions, the Blöchl projector augmented wave method (PAW) was employed within the frozen-core approximation [2] and the calculations utilized plane-wave basis sets. The generalized gradient approximation (GGA) of Perdew-Burke-Ernzerhof (PBE) [3] was selected as the exchange-correlation functional. For the 2D layers examined in this work, the lattice vector along the *z* direction was fixed to 20 Å to avoid the spurious interactions between periodic images. After a careful convergence test on the supercell, a 3 × 2 × 1 supercell with a 6 × 3 × 1 ***k***-mesh was adopted, which allowed us to explore the adsorption and related thermodynamical properties. For all relaxations, the self-consistence convergence criterion for electron iterations was set to 10^-6^ eV, and the ground-state structures were optimized by minimizing the Hellman-Feymann force until the norm of all the forces was less than 0.01 eV/Å.

## S2 Supplementary Figures and Tables


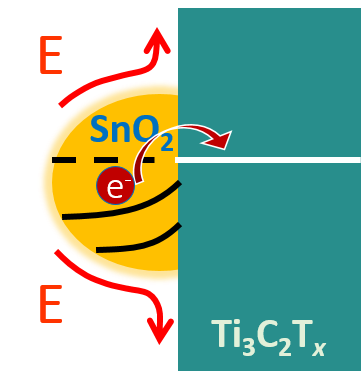


**Fig. S1** Illustration of the built-in electric field (BIEF) direction in the SnO_2_ nanoparticles and Ti_3_C_2_T*_x_* MXene plane formed heterostructure

Electrons transfer from SnO_2_ to Ti_2_C_2_T*_x_* MXene is driven by the difference in their work functions. This electron transfer persist until the Fermi level of both materials reach equilibrium. Consequently, there is an upward bending in the electron band of SnO_2_, leading to the establishment of the BIEF, which extends from the SnO_2_ side towards the MXene side


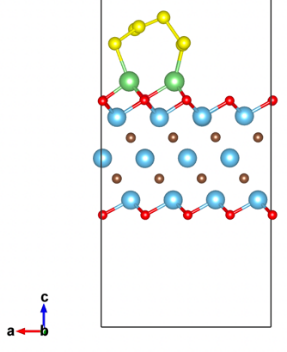

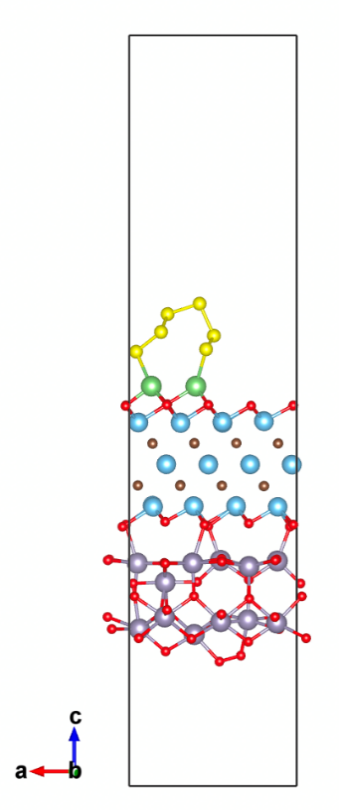

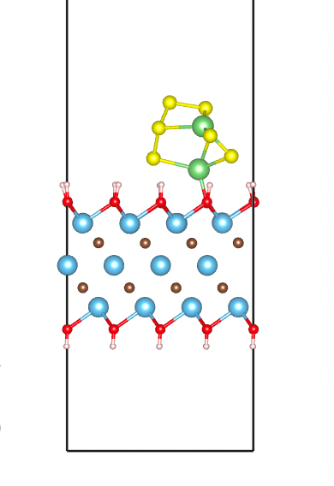

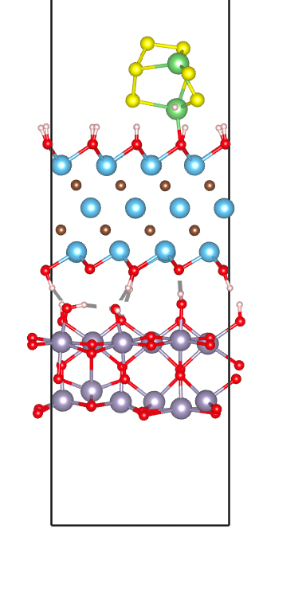


**Fig. S2** The side view of the structural models of MXO-Li_2_S_6_, MXO-SnO_2_-Li_2_S_6_, MXOH-Li_2_S_6_ and MXOH-SnO_2_-Li_2_S_6_, from the left to right

**Fig. S3** COOP of the Li-O bond analysis, in which the Fermi energy is set to 0 eV


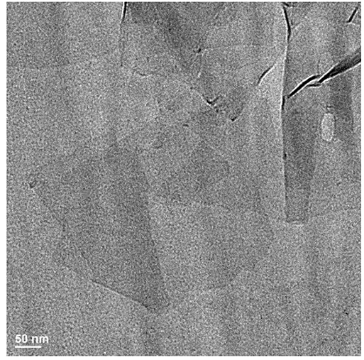


**Fig. S4** TEM of MXene nanosheets


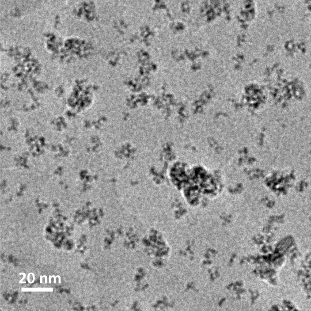


**Fig. S5** TEM image of SnO_2_@MX for the better identification of the aggregation


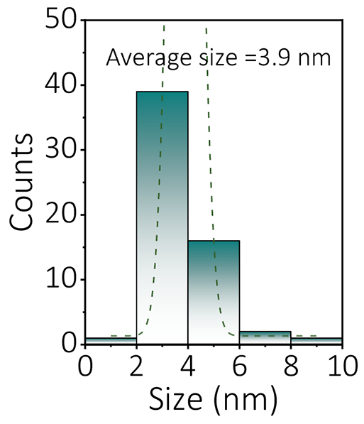


**Fig. S6** Size distribution of SnO_2_ nanoparticles


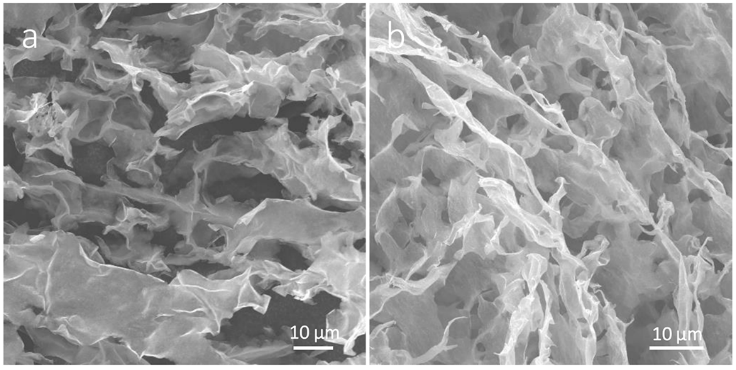


**Fig. S7** SEM images of freeze-dried **a**) MXene and **b**) SnO_2_@MXene powder. Both MXene and SnO_2_@MX exhibit similar morphologies, indicating that the fundamental two-dimensional properties of SnO_2_@MXene remain unchanged


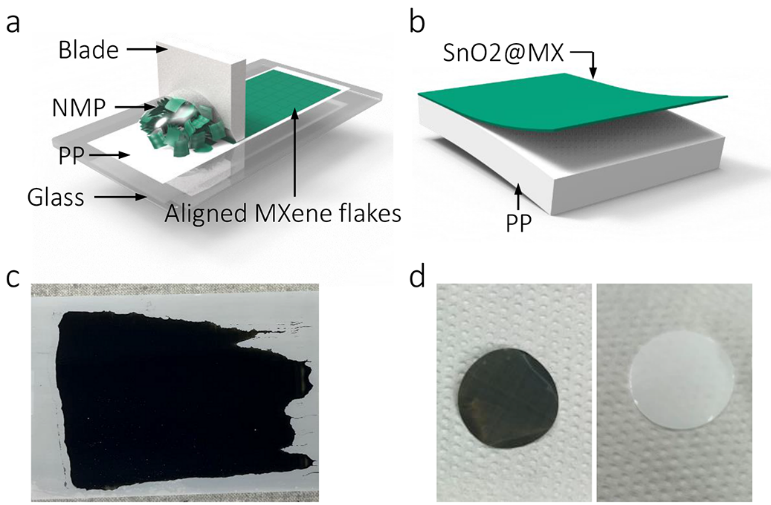


**Fig. S8** Schematic diagram of **a**) blade coating of SnO_2_@MX paste and **b**) SnO_2_@MX-PP separator. Photogragh of **c**) the coated film after blading and **d**) the obtained SnO_2_@MX-PP and PP separators


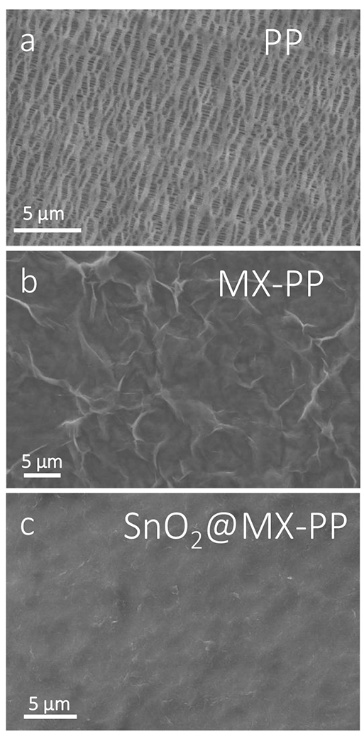


**Fig. S9** Top-view SEM images of as-prepared **a**) PP, **b**) MX-PP and **c**) SnO_2_@MX-PP


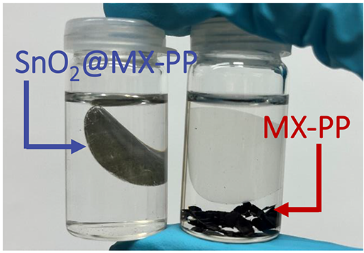


**Fig. S10** Photograph of SnO_2_@MX-PP and PP-MX in DOL solvent after vigorous shaking. The MX interlayer shattered into fragments, whereas the SnO_2_@MX-PP adhered firmly to the PP separator


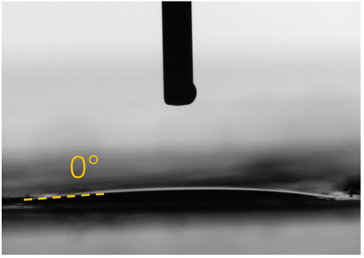


**Fig. S11** Contact angel between electrolyte and MX-PP separator


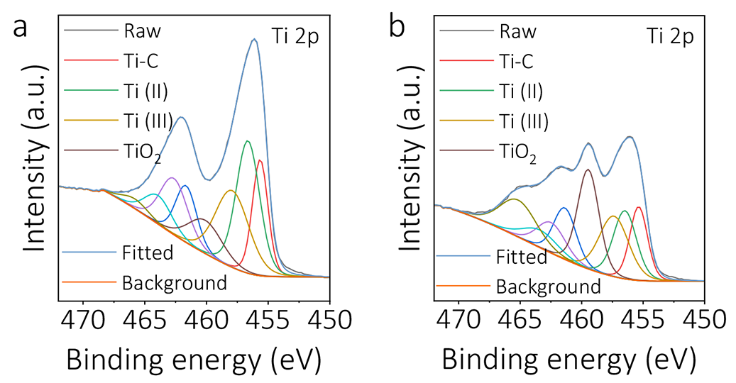


**Fig. S12** XPS Ti 2p spectra of **a**) MX and **b**) H-MX


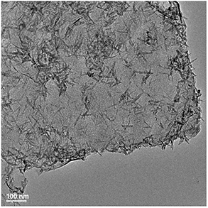


**Fig. S13** TEM image of H-MX


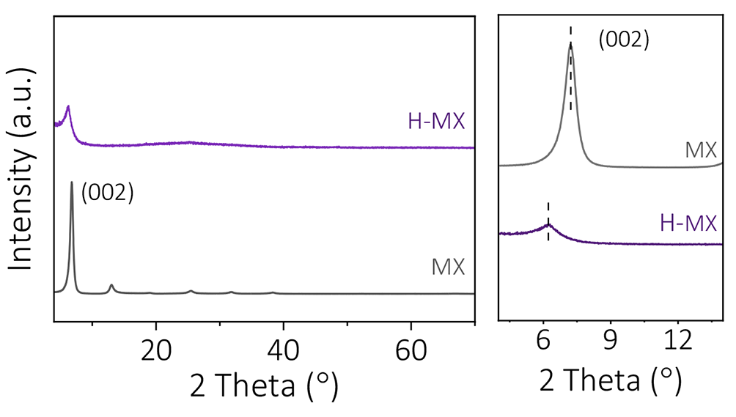


**Fig. S14** XRD spectrum comparison of MX and H-MX


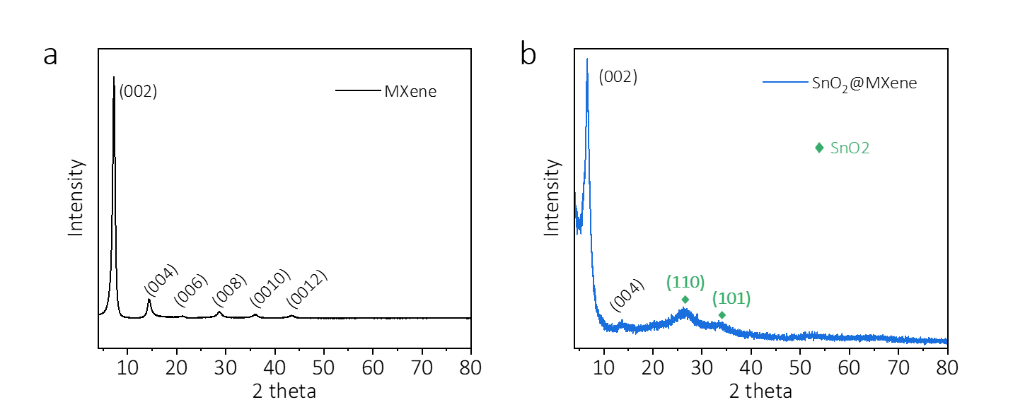


**Fig. S15** The detailed XRD pattern of MXene and SnO_2_@MX

From the detailed XRD pattern of MXene, we can see that besides the very prominent peak observed at 7-8°, several peaks are sequentially observed from 10 to 50°, including the most distinct peak located at 14-15°. As marked in the figure, the peak at 7-8° is the characteristic peak of MXene, representing the *d*-spacing between MXene layers, denoted as the (002) plane. The subsequent peaks are denoted as (004), (006), (008), (0010), and (0012) planes, representing the stacking in the [000*l*] direction. This indicates that the MXene material possesses a highly ordered layered structure. Ideally, the (002) peak corresponds to the basic unit of spacing between MXene layers, while peaks such as (004) and (006) are integer multiples of this distance, indicating the periodic repetition in the vertical direction of the layered structure. Moreover, the presence and clarity of these peaks are direct evidence of the high MXene crystal quality and the orderliness of the layers. In addition, although peaks for SnO_2_ are not observable in Figure 3c of the manuscript, by adjusting the vertical scale, we can still observe the (110) and (101) planes of SnO_2_ at 27° and 34° respectively.


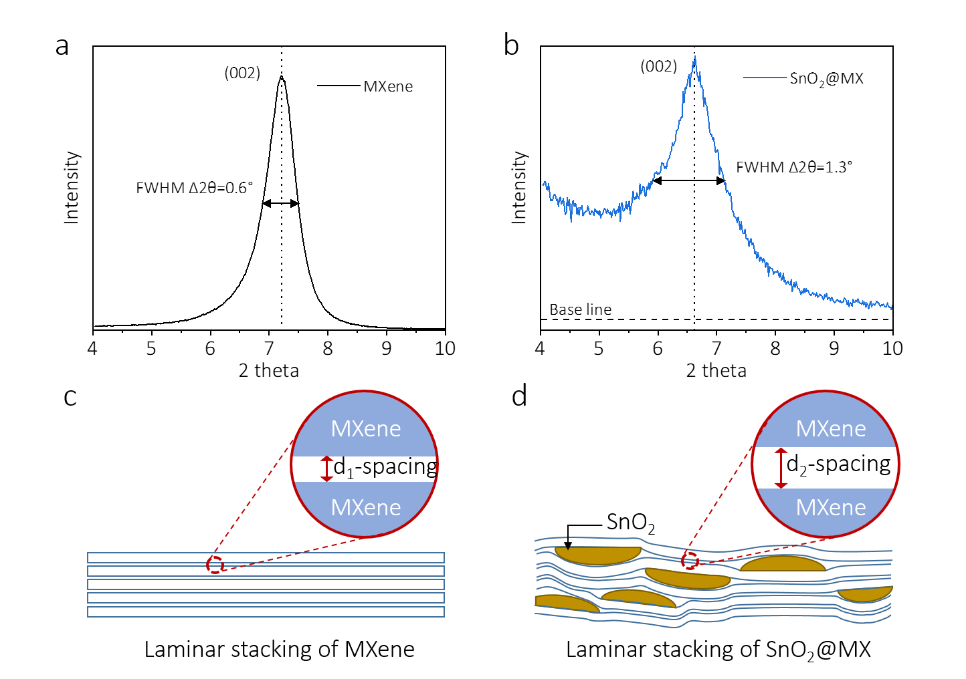


**Fig. S16** XRD patterns of a) MXene and b) SnO_2_@MX interlayer between 4-10°. Illustrative dia-grams of the d-spacing in laminar stacking for c) MXene and d) SnO_2_@MX

Intercalating nanoparticles into MXene leads to disordered stacking and an overall increase of interlayer spacing. This disruption of the originally orderly stacking results in a broader Full Width at Half Maximum (FWHM), as illustrated in Fig. S16a, b. It should be noted that the yielded *d*-spacing in SnO_2_@MX does not reflect the scale of nanoparticles, but the impact of nanoparticles on the original *d*-spacing, such as lattice distortion. As shown in Fig. c and d, the lattice distortion caused by the intercalation of SnO_2_ QDs leads to an increase in *d*-spacing relative to MXene. Therefore, the peak shift indicates that the intercalation of SnO_2_ QDs affects the stacking of MXene layers, resulting in an increase in *d*-spacing.


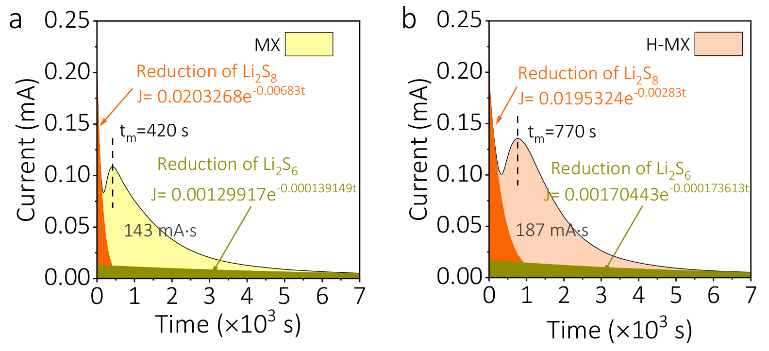


**Fig. S17** Precipitation tests of **a**) MX and **b**) H-MX electrodes. The response time of the peak current and the accumulated area of Li_2_S deposition, as simulated from the deposition curve, are highly correlated with Li_2_S nucleation and growth rate


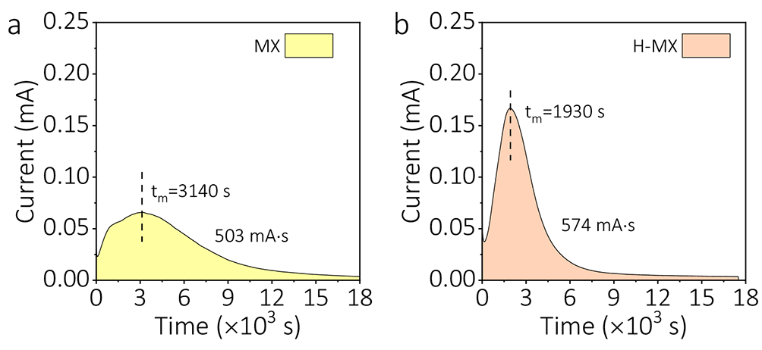


**Fig. S18** Dissolution tests of **a**) MX and **b**) H-MX electrodes


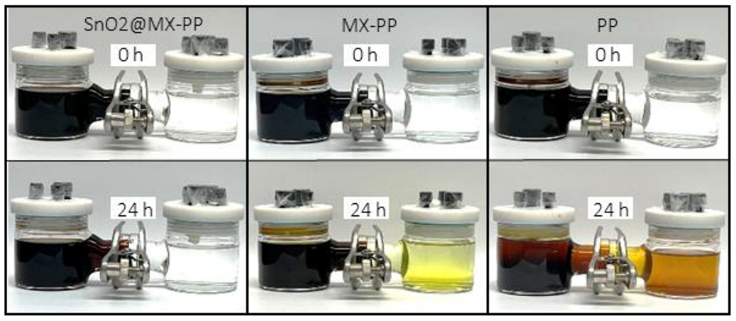


**Fig. S19** Digital photographs of LiPSs diffusion tests with different modified separators, where SnO_2_@MX-PP, MX-PP and PP were respectively inserted between two chambers containing Li_2_S_6_/DOL solution in one side and pure DOL solvent in the other side. It is evidenced from the color observed in the right chambers that the SnO_2_@MX-PP shows the least diffusion of LiPSs, followed by the MX-PP, and finally the PP separator


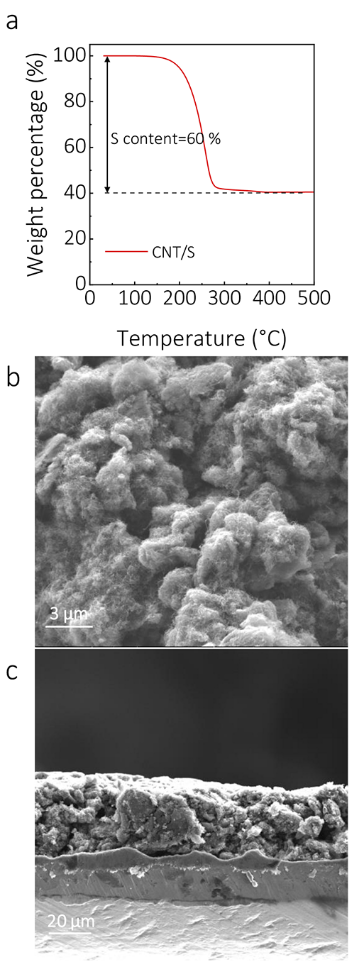


**Fig. S20** **a**) TGA of CNT/S composite. **b**) SEM of CNT/S composite. **c**) Cross-section SEM image of CNT/S cathode. The sulfur content in CNT/S cathode is 60%


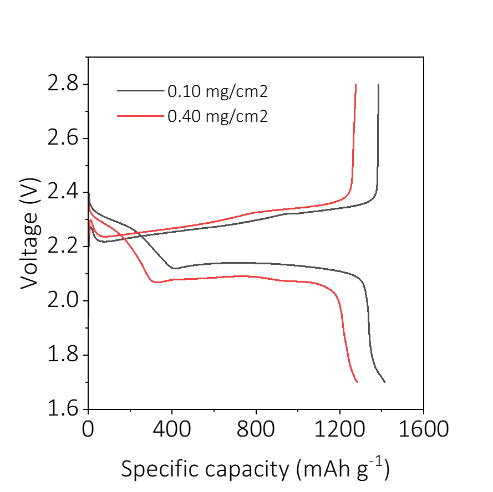


**Fig. S21** Charge-discharge curve of SnO_2_@MX-PP based cell at 0.05 C with various SnO_2_@MX loadings in the interlayer

An appropriate number of lithophilic sites in SnO_2_@MX interlayer facilitate Li^+^ diffusion efficiency. However, overly dense concentration or too strong interaction with the sites might slow down the movement of Li^+^ because it takes more energy for them to detach from these sites. As shown in Fig.S21, the electrochemical performance deteriorates with a thicker interlayer due to restricted Li^+^ transport.


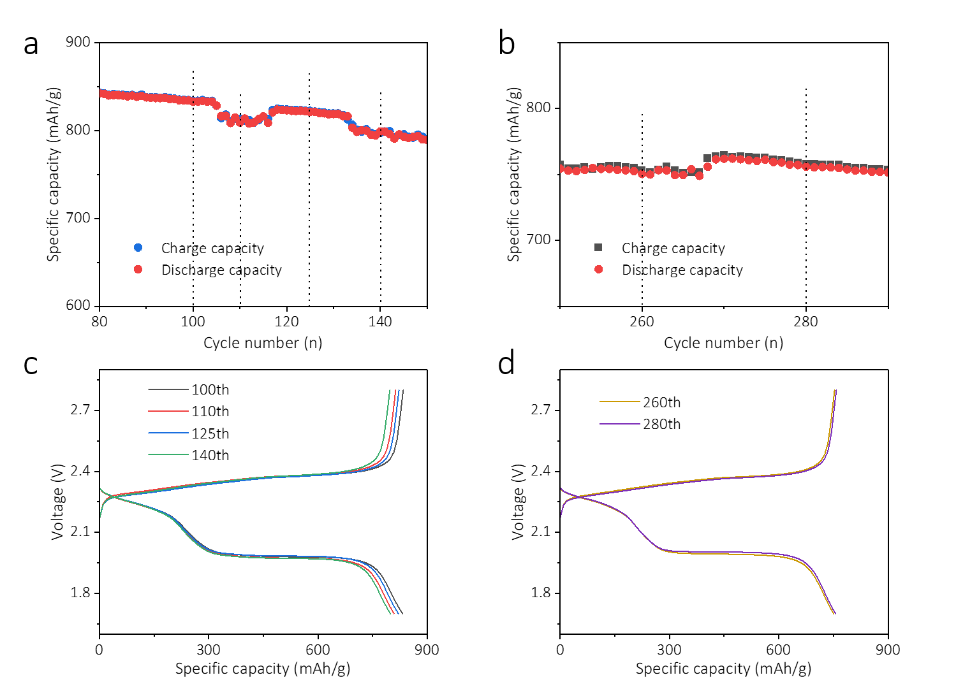


**Fig. S22** Detailed cyclic and charge-discharge curves of SnO_2_@MX-PP based cell at 2 C within **a**) 80-150 cycles and **b**) 250-290 cycles and **c, d**) the corresponding charge-discharge curves within these cycles

In Fig. 4g, minor fluctuations in capacity was observed for the SnO_2_@MX-PP, characterized by a cyclic pattern of capacity decrease to a lower level, recovery to the initial higher level after several cycles, a subsequent decrease, and eventual stabilization at a high level. The capacity fluctuations exhibit a variance of less than 3%, with no substantial changes in the shape of the charge-discharge curves, indicating that these minor fluctuations do not significantly impact the overall cyclic performance of the battery. We hypothesize that these fluctuations may be attributed to temporary structural changes in the electrode materials, such as minor cracks or detachment, which could slightly reduce the utilization efficiency of the active material. Nonetheless, the battery demonstrates inherent self-healing mechanisms that likely stabilize these changes after several cycles.


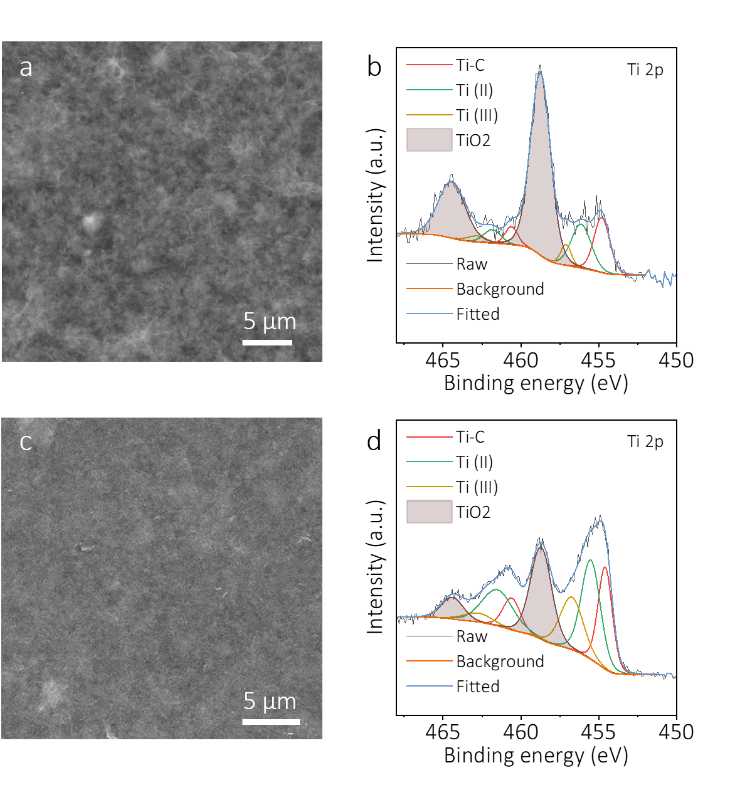
**Fig. S23** **a**) SEM and **b**) XPS of Ti spectra of SnO_2_@MX-PP after aging for approximately one year in air. **c**) SEM and **d**) XPS of Ti spectra of SnO_2_@MX-PP after cycling and aging for approximately one year in a Li-S cell

After being stored at room temperature in air for approximately one year, the surface morphology and the oxidation state of Ti in SnO_2_@MX-PP are illustrated in Fig. S23a and b. Notably, the TiO_2_ peak intensity is much stronger than the pristine SnO_2_@MX (Fig. 3b), indicating oxidation of the MXene in air. Fig. c and d display the morphology and Ti oxidation states of SnO_2_@MX-PP in the cell, which was extracted from a disassembled battery after undergoing cycling and aging within the cell for nearly a year. Although the TiO_2_ intensity is still stronger compared to pristine SnO_2_@MX, peaks attributed to MXene (Ti-C, Ti(II), and Ti(III)) are largely maintained, suggesting a certain level of chemical stability of SnO_2_@MX-PP within the battery. Furthermore, the surface in Fig. S23c is smoother than in Fig. S23a, also reflecting a lower degree of oxidation.


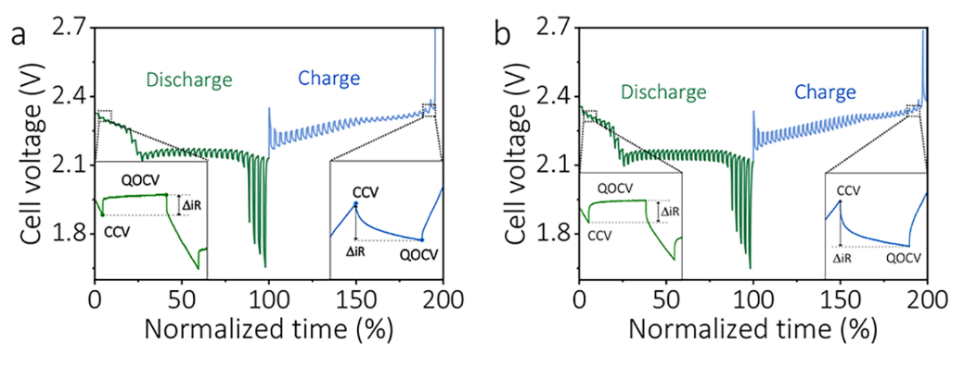


**Fig. S24** GITT tests of **a**) PP and **b**) MX-PP based Li-S cells


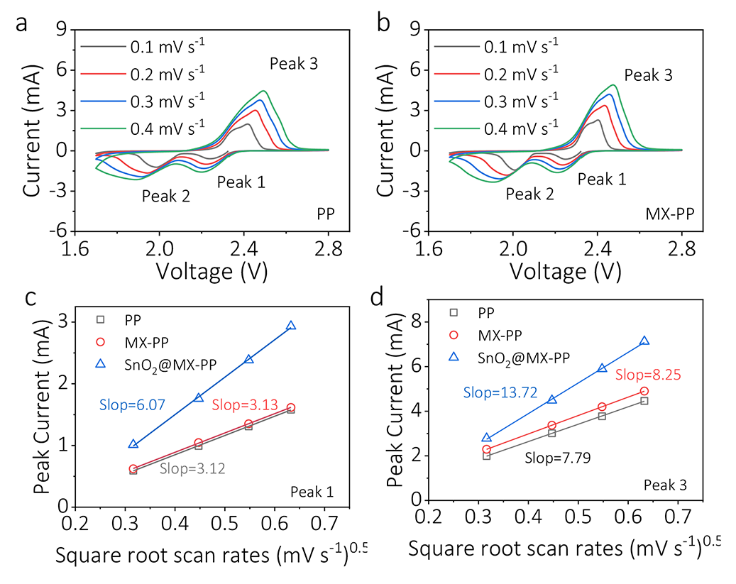


**Fig. S25** CV curves of the cells with **a**) PP and **b**) MX-PP separator at gradient scan rates. The plots of the **c**) peak 1 and **d**) peak 3 current versus the square root of scan rates with different separators


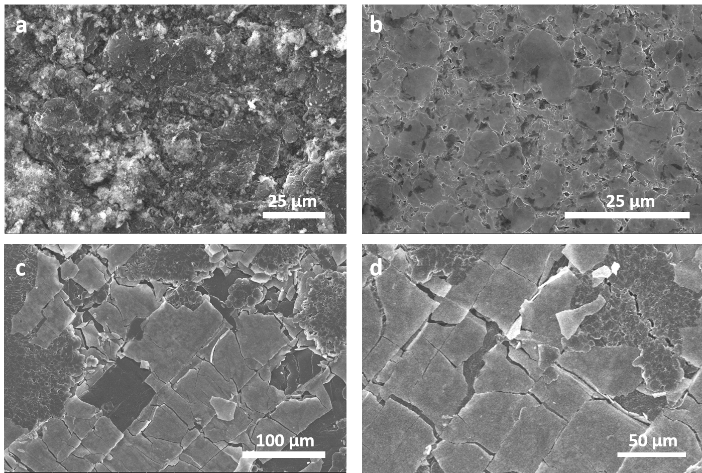


**Fig. S26** SEM image of Li anode surface after 400 cycles **a**) without PP separator, **b**) with SnO_2_@MX-PP separator. **c, d**) SEM image showing minor traces of SnO_2_@MX fragments remaining on the lithium anode surface after manual disassembly of the battery

Figure S26a reveals an irregular surface morphology of Li with PP separator, where the white, powdery areas indicate lithium dendrites. Fig. b shows a much smoother surface of Li anode with SnO_2_@MX interlayer, despite the presence of varying grain sizes. Additionally, Fig. c and d show minor traces of SnO_2_@MX fragments adhered to the Li surface after cell assembly, displaying signs of fracture. This confirms the high stiffness of SnO_2_@MX interlayer. The mor-phology beneath the SnO_2_@MX fractures is flatter. This vividly presents and confirms the me-chanical stress exerted by the interlayer controls the dendrite growth.


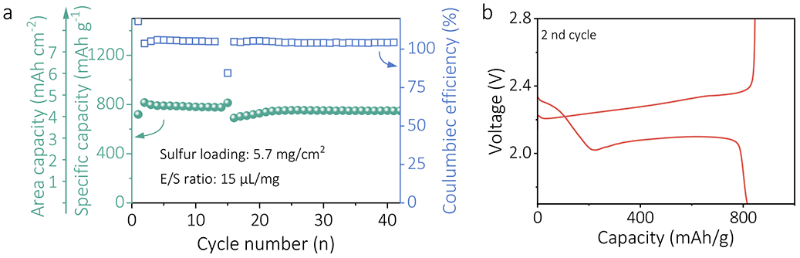


**Fig. S27** **a**) Cyclic performance of SnO_2_@MX-PP at 0.05 C with a sulfur loading of 5.7 mg/cm^2^ and E/S ratio of 15 μL/mg and **b**) the corresponding charge-discharge curves for the second cycle


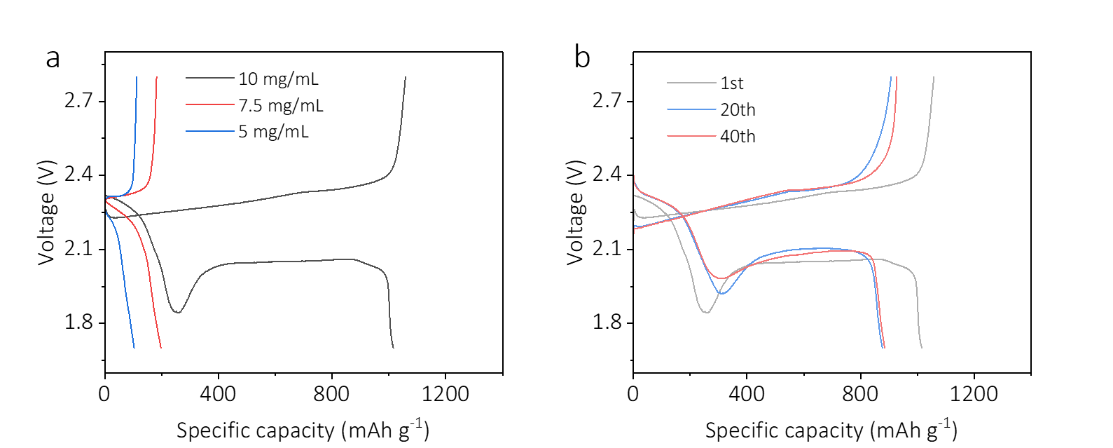


**Fig. S28** **a**) Electrochemical performance of high-loading CNT/S with ultrathin SnO2@MX interlayers at various E/S ratios. b) Charge-discharge curves for a 10 mg/mL E/S ratio with increased cycles

**Table S1** Adsorption energy (Eads) of Li_2_S_6_ on MXene and MXene-SnO_2_

| **Compounds** | MXO-Li_2_S_6_ | MXOH-Li_2_S_6_ | MXO-SnO_2_-Li_2_S_6_ | MXOH-SnO_2_-Li_2_S_6_ |
| --- | --- | --- | --- | --- |
| **E_ads_** | 2.33 | -1.38 | -0.99 | -2.57 |

**Table S2** The band levels of the metal *d*- and non-metal *p*- band centers are presented for MXOH, MXOH-SnO_2_, MXO and MXO-SnO_2_

|  | *p*-band center | *d*-band center |
| --- | --- | --- |
| MXOH | -5.65 eV | -1.24 eV |
| MXOH-SnO_2_ | -4.95 eV | -0.91 eV |
| MXOH | -5.65 eV | -1.47 eV |
| MXO-SnO_2_ | -4.22 eV | -1.30 eV |

**Table S3** Parameter of the equivalent circuit corresponding to EIS fitting result

| Separator based Li-S cell | R_Ω_ (Ω) | R_ct_ (Ω) |
| --- | --- | --- |
| PP | 4.58 | 73.59 |
| MX-PP | 2.8 | 58.9 |
| SnO_2_@MX-PP | 2.84 | 30.37 |

**Table S4** Comparison of high sulfur loading performance with recent reported works

| **Cathode**  **(sulfur loading)** | **Separator**  **(interlayer thickness)** | **E/S ratio** | **Initial Capacity (current rate)** | **Capacity retention (cycle numbers)** | **Refs.** |
| --- | --- | --- | --- | --- | --- |
| KB (Ketjen Black)/S  (2.3 mg/cm^2^) | MnN_3_OH@BNC/PP  (28 μm) | 15 μL/mg | ~1100 mAh/g  (0.2 C) | 894.5 mAh/g  (100 cycles) | [S4] |
| CNT/S  (5.9 mg/cm^2^) | SnSSe/rGO@PP  (9 μm) | 15 μL/mg | ~1186 mAh/g  (0.1 C) | ~780 mAh/g  (50 cycles) | [S5] |
| Super P/S  (10.7 mg/cm^2^) | Co/Co_0.85_Se@NC-PP  (7 μm) | 5.8 μL/mg | ~935 mAh/g  (0.05 C) | ~720 mAh/g  (40 cycles) | [S6] |
| CNT/S  (8.9 mg/cm^2^) | NC@CoNiFe/CNTs-PP  (15 μm) | 11.24 μL/mg | 999 mAh/g  (0.1 C) | 813.5 mAh/g  (100 cycles) | [S7] |
| KB/S  (3.27 mg/cm^2^) | HCCP-TBBA@CNTs-PP  (8 μm) | 10 μL/mg | 933 mAh/g  (0.2 C) | 829 mAh/g  (100 cycles) | [S8] |
| CNT/S  (7.06 mg/cm^2^) | NbB_2_/rGO/PP  (7.2 μm) | 10 μL/mg | ~600 mAh/g  (0.1 C) | 591 mAh/g  (40 cycles) | [S9] |
| KB/S  (6.0 mg/cm^2^) | NCZTC-PP  (7.6 μm) | 6 μL/mg | 866.7 mAh/g  (0.1 C) | ~700 mAh/g  (100 cycles) | [S10] |
| KB/S  (3.2 mg/cm^2^) | Co_3_Fe_7_-MXene-PP  (3 μm) | 4 μL/mg | ~580 mAh/g  (0.2 C) | ~530 mAh/g  (45 cycles) | [S11] |
| Active C/S  (3 mg/cm^2^) | g-DCN/Celgard separator  (8.3 μm) | 11.8 μL/mg | 1233 mAh/g  (0.1 C) | 933 mAh/g  (50 cycles) | [S12] |
| MoSe_2_-NSHC/S  (3.2 mg/cm^2^) | MoSe_2_-NSHC-PP  (7 μm) | 10 μL/mg | 1212 mAh/g  (0.1 C) | 807 mAh/g  (50 cycles) | [S13] |
| CNT/GO/S  (5 mg/cm^2^) | Fe-ZIF-8/PP  (8 μm) | 5 μL/mg | 517 mAh/g  (0.05 C) | ~500 mAh/g  (4 cycles) | [S14] |
| CNT/S  (3.8 mg/cm^2^) | H-TiN-Cu-CNFs/PP  (21 μm) | 10 μL/mg | 1144 mAh/g  (0.1 C) | 667 mAh/g  (100 cycles) | [S15] |
| CNT/S or KB/S  (5.6 mg/cm^2^) | DC/A O-CoVSe NPs/PP  (7.06 μm) | 8 μL/mg | 1027.4 mAh/g  (0.2 C) | 658.8 mAh/g  (100 cycles) | [S16] |
| Super P/S  (5.3 mg/cm^2^) | CoFe/NC@NC-PP  (19 μm) | Unknown | ~1000 mAh/g  (0.1 C) | 764 mAh/g  (100 cycles) | [S17] |
| CNT/S  (7.5 mg/cm^2^) | SnO_2_@MX-PP  (0.5 μm) | 10 μL/mg | 1015 mAh/g  (0.02 C) | 878 mAh/g  (50 cycles) | **Our work** |

**Supplementary References**

1. G. Kresse, J. Furthmuller, Efficient iterative schemes for ab initio total-energy calculations using a plane-wave basis set. Phys. Rev. B **54**, 11169-11186 (1996). <https://doi.org/10.1103/PhysRevB.54.11169>
2. G. Kresse, D. Joubert, From ultrasoft pseudopotentials to the projector augmented-wave method. Phys. Rev. B **59**, 1758-1775 (1999). <https://doi.org/10.1103/PhysRevB.59.1758>
3. J. P. Perdew, K. Burke, M. Ernzerhof, Generalized gradient approximation made simple. Phys. Rev. Lett. **77**, 3865-3868 (1996). <https://doi.org/10.1103/PhysRevLett.78.1396>
4. Y.R. Yan, N. Fu, W. Shao, T.T. Wang, Y. Liu, et al., Pinpointing the cl coordination effect on Mn-N_3_Cl moiety toward boosting reaction kinetics and suppressing shuttle effect in Li-s batteries. Small 2311799 (2024). <https://doi.org/10.1002/smll.202311799>
5. Z. Z. Yang, Z. D. Guo, X. Wang, W. Q. Lu, Q. Wang, et al., Bi-functional material snsse/rgo with anionic vacancies serves as a polysulfide shuttling blocker and lithium dendrite inhibitor. Energy Storage Mater. **67**, 103276 (2024). <https://doi.org/10.1016/j.ensm.2024.103276>
6. H. F. Xu, Q.B. Jiang, K.S. Hui, S. Wang, L.W. Liu, et al., Interfacial "double-terminal binding sites" catalysts synergistically boosting the electrocatalytic Li_2_S redox for durable lithium-sulfur batteries. ACS Nano **18**, 8839-8852 (2024). <https://doi.org/10.1021/acsnano.3c11903>
7. J.P. Shang, C. Ma, C.J. Zhang, W.W. Zhang, B.G. Shen, et al., Nitrogen-doped carbon encapsulated trimetallic conife alloy nanoparticles decorated carbon nanotube hybrid composites modified separator for lithium-sulfur batteries. J. Energy Storage **82**, 110552 (2024). <https://doi.org/10.1016/j.est.2024.110552>
8. X.X. Dong, W.W. Gu, X. Tong, G.Q. Liu, J. Sun, et al., In situ growth strategy to construct "four-in-one" separators with functionalized polyphosphazene coatings for safe and stable lithium-sulfur batteries. Small 2311471 (2024). <https://doi.org/10.1002/smll.202311471>
9. Y.J. Li, Z.Z. Wang, H.F. Gu, H.P. Jia, Z.Y. Long, et al., Niobium boride/graphene directing high-performance lithium-sulfur batteries derived from favorable surface passivation. ACS Nano **18**, 8863-8875 (2024). <https://doi.org/10.1021/acsnano.3c12076>
10. X.P. Wu, R.J. Xie, D.P. Cai, B. Fei, C.Q. Zhang, et al., Engineering defect-rich bimetallic telluride with dense heterointerfaces for high-performance lithium-sulfur batteries. Adv. Funct. Mater. 2315012 (2024). <https://doi.org/10.1002/adfm.202315012>
11. Z.R. Wang, H.Y. Jiang, C.L. Wei, K.D. Tian, Y. Li, et al., Ultrasmall cofe bimetallic alloy anchored on fluoride-free mxene by one-pot etching strategy for the barrier-adsorption-catalyst functions of polysulfides in lithium-sulfur batteries. Adv. Funct. Mater. 2315178 (2024). <https://doi.org/10.1002/adfm.202315178>
12. W.H. Sun, L.Y. Xu, Z.H. Song, H. Lin, Z.Q. Jin, et al., Coordination engineering based on graphitic carbon nitrides for long-life and high-capacity lithium-sulfur batteries. Adv. Funct. Mater. 2313112 (2023). <https://doi.org/10.1002/adfm.202313112>
13. Q.D. Sheng, H. Liu, Y.N. Liu, B. Jin, M.Y. Cui, et al., Functional separator with 1 T/2H-MoSe_2_ nanosheets decorated nitrogen and sulfur Co-doped mesoporous hollow carbon spheres for high-performance li-s batteries. Chem. Eng. J **476**, 146880 (2023). <https://doi.org/10.1016/j.cej.2023.146880>
14. R. Razaq, M.M.U. Din, D.R. Småbråten, V. Eyupoglu, S. Janakiram, et al., Synergistic effect of bimetallic mof modified separator for long cycle life lithium-sulfur batteries. Adv. Energy Mater. **14**, 2302897 (2024). <https://doi.org/10.1002/aenm.202302897>
15. Y.Y. Xiang, L.Q. Lu, F. Yan, D. Sengupta, P. Rudolf, et al., Tuning the crystallinity of titanium nitride on copper-embedded carbon nanofiber interlayers for accelerated electrochemical kinetics in lithium-sulfur batteries. Carbon Energy e450 (2024). <https://doi.org/10.1002/cey2.450>
16. P.C. Tan, Y. Yin, D.P. Cai, B. Fei, C.Q. Zhang, et al., Oxygen-incorporated crystalline/amorphous heterophase cobalt vanadium selenide nanoplates with dense interfacial sites for robust lithium-sulfur batteries. J. Mater. Chem. A **12**, 3711-3721 (2024). <https://doi.org/10.1039/d3ta07189h>
17. H.L. Wei, Y.L. Gong, C.M. Gao, Z.X. Chen, Z.Y. Zhou, et al., The CoFeNC@NC catalyst with numerous surface cracks bidirectionally catalyzes the conversion of polysulfides to accelerate the reaction kinetics of lithium-sulfur batteries. Small **20**, 2304531 (2024). <https://doi.org/10.1002/smll.202304531>
